# The long and short non-coding RNAs modulating EZH2 signaling in cancer

**DOI:** 10.1186/s13045-022-01235-1

**Published:** 2022-03-02

**Authors:** Sepideh Mirzaei, Mohammad Hossein Gholami, Kiavash Hushmandi, Farid Hshemi, Amirhossein Zabolian, Israel Canadas, Ali Zarrabi, Noushin Nabavi, Amir Reza Aref, Francesco Crea, Yuzhuo Wang, Milad Ashrafizadeh, Alan Prem Kumar

**Affiliations:** 1 Department of Biology, Faculty of Science, Islamic Azad University, Science and Research Branch, Tehran, Iran; 2 Faculty of Veterinary Medicine, Kazerun Branch, Islamic Azad University, Kazerun, Iran; 3 Department of Food Hygiene and Quality Control, Division of Epidemiology and Zoonoses, Faculty of Veterinary Medicine, University of Tehran, Tehran, Iran; 4 Department of Comparative Biosciences, Faculty of Veterinary Medicine, University of Tehran, 1417466191 Tehran, Iran; 5 Department of Orthopedics, School of Medicine, 5th Azar Hospital, Golestan University of Medical Sciences, Gorgan, Golestan Iran; 6 Blood Cell Development and Function Program, Fox Chase Cancer Center, Philadelphia, PA USA; 7 Department of Biomedical Engineering, Faculty of Engineering and Natural Sciences, Istinye University, Istanbul, 34396 Turkey; 8 Department of Urological Sciences and Vancouver Prostate Centre, University of British Columbia, Vancouver, BC V6H3Z6 Canada; 9 Belfer Center for Applied Cancer Science, Dana-Farber Cancer Institute, Harvard Medical School, Boston, MA USA; 10 Department of Translational Sciences, Xsphera Biosciences Inc., Boston, MA USA; 11 Cancer Research Group-School of Life Health and Chemical Sciences, The Open University, Walton Hall, Milton Keynes, MK7 6AA UK; 12 Faculty of Engineering and Natural Sciences, Sabanci University, Orta Mahalle, Üniversite Caddesi No. 27, Orhanlı, Tuzla, Istanbul 34956 Turkey; 13 Cancer Science Institute of Singapore and Department of Pharmacology, Yong Loo Lin School of Medicine, National University of Singapore, Singapore, 117599 Singapore; 14 NUS Centre for Cancer Research (N2CR), Yong Loo Lin School of Medicine, National University of Singapore, Singapore, Singapore

**Keywords:** EZH2, MiRNA, LncRNA, CircRNA, SiRNA, ShRNA, Cancer therapy

## Abstract

Non-coding RNAs (ncRNAs) are a large family of RNA molecules with no capability in encoding proteins. However, they participate in developmental and biological processes and their abnormal expression affects cancer progression. These RNA molecules can function as upstream mediators of different signaling pathways and enhancer of zeste homolog 2 (EZH2) is among them. Briefly, EZH2 belongs to PRCs family and can exert functional roles in cells due to its methyltransferase activity. EZH2 affects gene expression via inducing H3K27me3. In the present review, our aim is to provide a mechanistic discussion of ncRNAs role in regulating EZH2 expression in different cancers. MiRNAs can dually induce/inhibit EZH2 in cancer cells to affect downstream targets such as Wnt, STAT3 and EMT. Furthermore, miRNAs can regulate therapy response of cancer cells via affecting EZH2 signaling. It is noteworthy that EZH2 can reduce miRNA expression by binding to promoter and exerting its methyltransferase activity. Small-interfering RNA (siRNA) and short-hairpin RNA (shRNA) are synthetic, short ncRNAs capable of reducing EZH2 expression and suppressing cancer progression. LncRNAs mainly regulate EZH2 expression via targeting miRNAs. Furthermore, lncRNAs induce EZH2 by modulating miRNA expression. Circular RNAs (CircRNAs), like lncRNAs, affect EZH2 expression via targeting miRNAs. These areas are discussed in the present review with a focus on molecular pathways leading to clinical translation.

## Introduction

Over the past decades, attention has been directed towards revealing the role of signaling networks in cancer, as this disease causes high mortality and morbidity worldwide [1]. It is believed that abnormal proliferation and metastasis of cancer cells result from alterations in molecular pathways [2–4]. In fact, these tumor-promoting molecular pathways drive cancer progression via activating positive factors for cancer survival [5–7]. In contrast, tumor-suppressor pathways sensitize cancer cells to death and prevent their progression and migration [8]. Due to progress in sequencing and bioinformatics, such molecular pathways have been identified and by continuing research, other novel signaling networks with potential roles in cancer progression/inhibition are revealed [9–11]. The importance of revealing such molecular pathways paves the way for developing novel therapeutics for effective cancer treatment. These therapeutics can be based on designing genetic tools for targeting molecular pathways or using small molecules as drugs for suppressing cancer progression [12–15]. Furthermore, plant derived-natural products have also demonstrated capacity in targeting molecular pathways for cancer therapy [16–19]. According to newly published article by Siegel and colleagues, cancer is still a major challenge for public health and more research should be directed towards basic and clinical understanding of cancer [20].

In the present review, our aim is to focus on a special signaling pathway known as enhancer of zeste homolog 2 (EZH2) in cancer, and its regulation by major upstream mediators, called non-coding RNAs (ncRNAs). The regulatory impact of these ncRNAs on EZH2 signaling, both induction and inhibition are discussed. Furthermore, to ease the understanding of the miRNA roles, we divided the sections based on cancer types. Each section is discussed with a focus on molecular pathways, and to pave the way for clinical translation, the role of these signaling networks, related biomarkers, and their application in clinic are discussed.

## EZH2 signaling in cancer

### Signaling pathways

There is a large protein family, known as Polycomb Repressive Complexes (PRCs) capable of modifying lysine residues on histones [21, 22]. In mammals, there are two primary PRCs including PRC1 and PRC2. EZH2 is a member of PRCs that exerts functional roles in cells [23]. In PCR2 complex, EZH2 is considered as a catalytic subunit and is responsible for methyltransferase activity. It is noteworthy that EZH proteins can be phosphorylated. However, there are differences among them. The phosphorylation of EZH1 results in its degradation and significantly diminishes its function [24, 25]. The regulatory impact of EZH2 on other genes is related to its impact on histone H3 lysine 27 (H3K27me3). H3K27me3 can be identified by WD40-repeat domain of EED, contributing to the spread of H3K27me3 in genome through the formation of feedforward loop. As a catalytic subunit of PRC2, EZH2 suppresses the transcription of target genes via triggering the trimethylation (Lysine-27) of H3K27me [26]. By regulating gene transcription, EZH2 participates in biological mechanisms including cell fate, cell lineage specification and tumorigenesis [27–30]. EZH2 can also affect cell cycle progression, autophagy, apoptosis, DNA damage repair, and cellular senescence [31–33]. Furthermore, EZH2 can interact with non-histone targets or directly interact with proteins to influence downstream targets in a PRC2-independent manner [34–36]. Overall, there are three different actions for EZH2 including PRC2-dependent H3K27m3, PRC2-dependent non-histone protein methylation, and PRC2-independent gene transactivation [37].

**Fig. 1:**
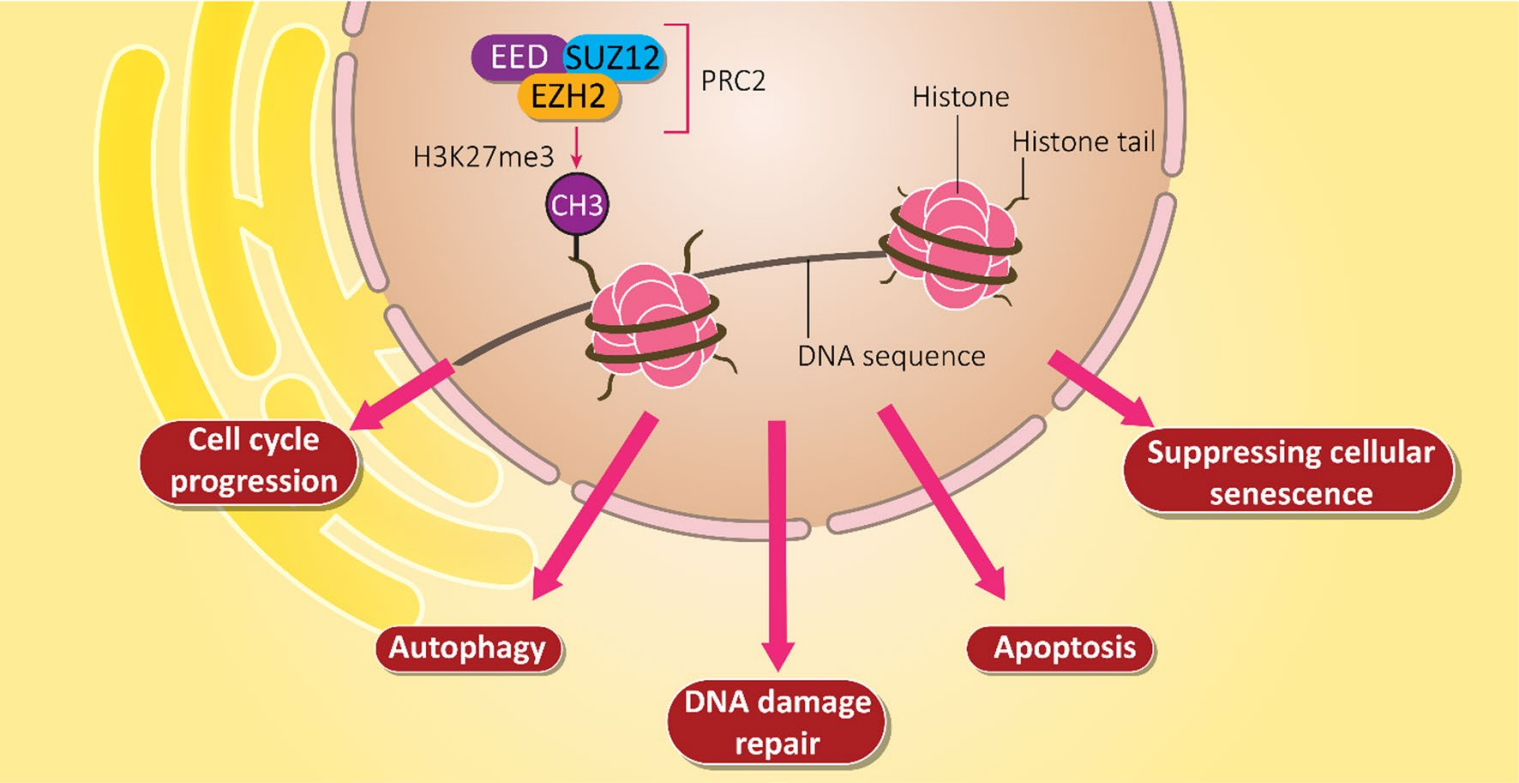
EZH2 signaling and its role in regulating downstream signaling pathways. Epigenetic regulation of molecular mechanisms in cells such as DNA repair, cell cycle, apoptosis, autophagy and senescence are regulated by EZH2, showing vital functions of this signaling pathway in cells

Structurally, *EZH2* gene is located on chromosome 7q35 and contains 20 exons with 746 amino acid residues [38]. It has been reported that EZH2 has five distinct domains that are responsible for its regulatory effects. These domains include EED-interaction domain (EID), Domain I and Domain II cysteine-rich domain (CXC domain), C-terminal suppressor of variegation 39 enhancer of zeste, and trithorax domain (SET domain) [39, 40]. Each domain has its own activities. For instance, SET domain cooperates with CXC domain N-terminal for histone methyltransferase activity of EZH2 through its N-terminal domains [39]. Figure 1 demonstrates role of EZH2 in regulation of vital biological mechanisms in cell.

### The roles of EZH2 in cancer

With respect to the role of EZH2 in regulation of biological mechanisms, studies have focused on revealing its role in cancer. For exerting its tumorigenesis impact, we need to consider EZH2 stability. It has been reported that protein arginine methyltransferase 1 (PRMT1) can promote EZH2 stability via its methylation. On the other hand, EZH2 enhances breast cancer metastasis via epithelial-to-mesenchymal transition (EMT) induction [41]. It is noteworthy that genome stability is affected by EZH2 in cancer cells. EZH2 overexpression is associated with instability of ribosomal DNA and an increase in ribosome synthesis that is in favor of increasing proliferation and invasion of breast cancer cells [42]. Therefore, EZH2 stabilization leads to cancer progression and silencing EZH2 diminishes cancer proliferation and metastasis [43]. It was showed that EZH2 stimulates EMT and leads to an increase in cancer metastasis. It seems that EZH2 can affect metabolism of cancer cells in increasing their growth. EZH2 uses its methyltransferase activity to silence expression of aldehyde oxidase (AOX1). These results in the activation of tryptophan-kynurenine pathway that subsequently promotes NADP levels to provide cancer growth [44]. It is noteworthy that EZH2 can regulate genes involved in cell cycle progression of cancer cells [45]. The Yes-associated protein (YAP) activates EZH2 to transcriptionally suppress expression level of p27, as a cell cycle kinase inhibitor [46]. EZH2 signaling can affect immune cells in favor of cancer progression. In this way, EZH2 stimulates chemokine ligand 5 (CCL5) to provide macrophage chemotaxis, leading to metastasis of lung cancer cells [47]. EZH2 also regulates the response of cancer cells to therapy. Low expression of EZH2 mediates better responses to chemotherapy compared to high expression. Additionally, EZH2 can be considered as a prognostic factor, so that low expression of EZH2 leads to a good prognosis and improve progression-free survival rate of ovarian cancer patients [48]. Furthermore, suppressing EZH2 signaling can prevent the development of drug resistance in small cell lung cancer [49].

Noteworthy, EZH2 signaling can regulate response of cancer cells to immunotherapy. The checkpoint inhibitors are commonly utilized in prostate cancer therapy. It has been reported that suppressing EHZ2 signaling enhances number of M1 macrophages and induces infiltration of CD8 + T cells, increasing response of prostate cancer cells to checkpoint inhibitors [50]. Furthermore, knock-down of EZH2 elevates IFN-γ-mediated cytokines, confirming modulatory impacts of EZH2 signaling on immune responses [51]. Clinical studies have confirmed the role of EZH2 in cancer prognosis. For instance, moderate and high expression levels of EZH2 are associated with unfavorable prognosis of patients with ovarian cancer [52]. Overall, EZH2 is a critical player in cancer [53–56] and can affect proliferation, metastasis, and therapy response of cancer cells. However, there are studies showing that EZH2 can also function as a tumor-suppressor. For instance, SOX4 overexpression is in favor of breast cancer progression and EZH2 reduces its expression by binding to the promoter, leading to a decrease in invasion and migration of breast cancer cells [57]. Furthermore, EZH2 has been shown to suppress immune evasion of hepatocellular carcinoma cells by recruiting H3K27me3 to promoter and negatively affect programmed death-ligand 1 (PD-L1) expression [58]. Table 1 provides a summary of EZH2’s role in different cancers.

**Table 1:** A summary of EZH2’s role in different cancers

Cancer type	Signaling network	Remarks	References
Breast cancer	EZH2/PP2A	EZH2 reduces expression level of PP2A via triggering histone modification Conferring resistance to HER2 inhibitors	[59]
Breast cancer	PRMT1/EZH2	Tumor-associated macrophages stimulate PRMT1 expression to enhance EZH2 stability and expression, leading to breast cancer invasion	[60]
Triple-negative breast cancer	EZH2/DLC1	Curcumin impairs metastasis and proliferation of cancer cells Curcumin induces apoptosis Reducing EZH2 expression and subsequent upregulation of DLC1	[61]
Non-small cell lung cancer	EZH2/TGFBR2	Synergistic impact between EZH2 and YAP/TAZ in transcription repression of TGFBR2 and promoting cancer progression	[62]
Lung cancer	–	Association of EZH2 overexpression with cancer proliferation, metastasis, and therapy resistance Providing poor prognosis	[63]
Colorectal cancer	DUXAP8/EZH2/EMT	Enhancing metastasis of cancer cells via EMT induction Activation of EZH2 by DUXAP8 is vital for EMT stimulation	[64]
Colon cancer	–	Overexpression of EZH2 in colon cancer compared to normal colonic mucosa Reduced tumor differentiation, and lymph node metastasis Association with lower survival Therefore, EZH2 can be considered as a prognostic factor	[65]
Ovarian cancer	EZH2/DAB2IP	DAB2IP overexpression suppresses cancer stem cell features in ovarian cancer EZH2 down-regulates DAB2IP expression to induce Wnt signaling, leading to ovarian cancer progression	[66]
Gastric cancer	EZH2/Rho/ROCK/EMT	EZH2 promotes cancer metastasis via inducing Rho/ROCK-mediated EMT Diosgenin and GSK126 synergistically down-regulate EZH2 in suppressing cancer metastasis	[67]
Prostate cancer	AR/EZH2	The interaction between AR and EZH2 leads to prostate cancer progression EZH2 inhibition enhances anti-tumor activity of metformin	[68]

## EZH2 inhibitors: an overview

In according to critical role of EZH2 signaling in vital biological mechanisms and modulating cancer progression as well as affecting immune system, significant efforts have been made in developing novel EZH2 inhibitors. Different kinds of EZH2 inhibitors have been developed and some of them are being applied in clinical trials for treatment of patients [69–73]. The S-adenosylmethionine (SAM)-competitive inhibitors are among the common EZH2 inhibitors in cancer therapy. The GSK126 is such compound that suppresses methyltransferase activity of EZH2 and diminishes H3K27me3 levels. These activities pave the way for stimulating silenced PRC2 downstream targets and preventing cancer growth in xenografts [74]. The EED226 is another EZH2 inhibitor capable of repressing PRC2 activity. There is a binding site on the H3K27me3, known as EED, that EED226 can bind to it, triggering conformational alterations and inhibiting PRC2 activity [75]. In addition to pre-clinical experiments, clinical studies have also evaluated role of EZH2 inhibitors in treatment of cancer patients. The intravenous administration of GSK1816126 demonstrates moderate anti-tumor activity in solid tumors and lymphoma [76]. The previous clinical trial was in phase I and highlighted that low half-life survival of EZH2 inhibitors limits their tumor-suppressor activity. Therefore, methods for targeted delivery of EZH2 inhibitors such as nanotechnological approaches can be developed in near future.

The first small molecule with capacity of EZH2 inhibition was carbocyclic adenosine analog 3-deazaneplanocin (DZNep) that was derived from neplanocin-A as a natural antibiotic [77]. However, DZNep lacks specificity and selectivity and it may negatively affect physiological processes such as regeneration [78]. Furthermore, DZNep had poor bilability and low half-life due to its hydrophilic nature [79]. Therefore, more selective EZH2 inhibitors have been developed. The EPZ005687 was developed as a high selective and potent small molecule capable of EZH2 inhibition. The EZP005687 demonstrated potential in preventing H3K27 methylation in a concentration-dependent manner and suppressed progression of various tumors including lymphoma, breast and prostate tumors [80]. The GSK126 can affect both mutant and WT EZH2 with high selectivity and it prevents the growth of lymphoma in mice and currently, it has been introduced to clinic (phase I) for treatment of lymphoma [81–83]. GSK343 is also an EZH2 inhibitor, but it has a different structure from GFSK126 and instead of indole, GSK343 has indazole nucleus [84].

It has been reported that EZH2 inhibitors are effective in suppressing metastasis and neovascularization in human tumors. The clinical aspect of EZH2 inhibitors is also attributed to improving ability of oncologists in suppressing metastasis and angiogenesis in cancer. Interestingly, tumor cells respond more likely to EZH2 inhibitors and they have been applied in treatment of colon cancer (stage II and III). A similar approach can be utilized in treatment of others cancers such as breast and gastric cancers. Furthermore, EZH2 inhibitors can be utilized as adjuvant in synergistic cancer therapy in clinical course. As out knowledge in oncology and EZH2 signaling enhances, more novel therapeutics for EZH2 targeting are introduced. Furthermore, since various complicated signaling networks are involved in cancer progression, EZH2 inhibitors and other anti-cancer agents should be used in combination in effective cancer therapy [85].

The administration route of EZH2 inhibitors in clinical course is also different. For instance, EPZ6438 and CPI-1205 are administered via oral route, while GSJ126 is administered via intravenous route [86]. There are some limitations associated with EZH2 inhibitors that should be considered for their clinical application. The poor efficacy, large molecular weight and poor bioavailability are most important drawbacks of EZH2 inhibitors [87]. For instance, TAZVERIK™ is a commercial EZH2 inhibitor that should be used with dose of 800 mg twice daily. Recently, efforts have been made in developing covalent inhibitors for EZH2 in cancer therapy. These kinds of inhibitors develop covalent bonds with EZH2 and they have improved pharmacodynamic duration. Therefore, there is no need of continuous exposure to EZH2 inhibitors [88]. Increasing evidence has shown benefits of covalent EZH2 inhibitors including high potency, good pharmacokinetics and unique selectivity [89–91]. However, a few covalent EZH2 inhibitors have been developed. For instance, GNA002 can covalently bind to EZH2 for inhibiting its function [92]. It is worth mentioning that covalent EZH2 inhibitors have also their drawbacks such as complex structure and low bioavailability that limits their clinical application [93]. Hence, more investigation should be made in developing novel and effective EZH2 inhibitors. Table 2 provides a summary of EZH2 inhibitors.

**Table 2:** An overview of EZH2 inhibitors based on pre-clinical and clinical studies

EZh2 inhibitor	In vitro/in vivo/clinical trial	Remarks	References
GSK2816126	Clinical trial (phase I)	Preventing the progression of solid tumors and lymphoma Exerting mild anti-tumor activity Low half-life restricts its anti-tumor activity Intravenous administration	[76]
GSK126	In vitro (DLBCL cell line) In vivo (xenografts)	Preventing methyltransferase activity of EZH2 Decreasing H2K27me3 levels Stimulating expression of PCR2 target genes	[74]
EED226	In vivo (human lymphoma xenograft tumors)	Triggering conformational changes in EED site of H3K27me3 Suppressing PRC2 activity Preventing tumor growth	[75]
GSK926 GSK343	In vitro (HCC1806 breast cancer cells)	Reducing nuclear H3K27me3 levels in a concentration-dependent manner Acting like other SAM compounds in suppressing EZH2 activity	[94]
EPZ-6438	In vitro (lymphoma cells) In vivo (EZH2-mutant NHL xenograft-bearing mice)	Acting in a time- and concentration-dependent manner Preventing lysine 27 methylation of H3K27me3 Suppressing EZH2 signaling Exerting anti-tumor activity	[95]
SAH-EZH2 (a peptide)	In vitro (MLL-AF9 leukemia cells)	Proliferation inhibition Inducing monocyte-macrophage differentiation Inhibiting EZH2 signaling by impairing EZH2-EED complex	[96]
AZD9291	In vitro (lymphoma and breast cancer cells)	Inhibiting PRC2 activity by disrupting EZH2-EED interaction Reducing EZH2 expression at mRNA and protein levels via miRNA-34a overexpression	[97]
Astemizole	In vitro (SU-DHL6, Toledo, DB, SU-DHL4, and Pfeiffer lymphoma cell lines)	Suppressing growth of cancer cells Inhibiting EZH2 signaling via preventing interaction between PRC2 and EZH2-EED complex	[98]
Wedelolactone	In vitro (HepG2, K562 and 293T cells)	Binding to EED and inhibiting EED and EZH2 interaction Mediating PRC2 degradation Suppressing cancer proliferation	[99]

## Non-coding RNAs and EZH2 signaling

### MiRNAs and EZH2 signaling

**Fig. 2:**
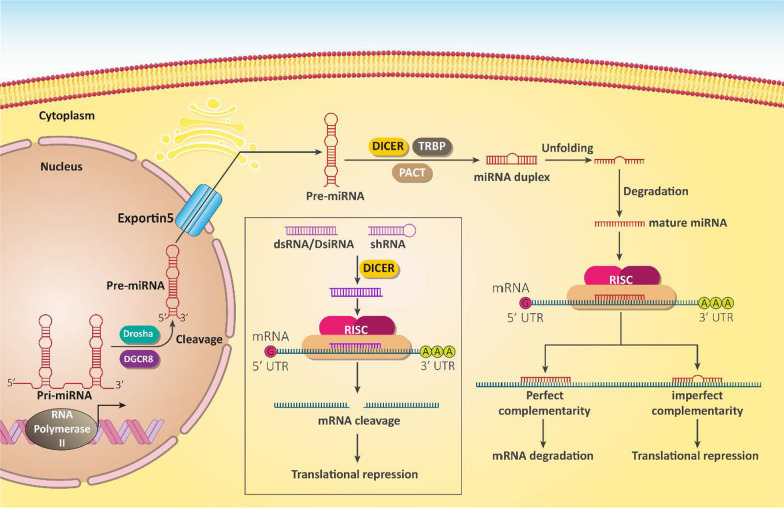
The biogenesis route and functions of miRNAs, siRNA and shRNA in cells [110, 111]

In 1993, the discovery of microRNAs (miRNAs) improved our knowledge of biological mechanisms and their regulations in cells [100–103]. These short ncRNAs have a length of 22 nucleotides and can bind to complementary target messenger RNA (mRNA) via 3′-untranslated region (3′-UTR) [104]. Before targeting mRNA, miRNAs form a complex with Argonaute protein to be embedded in RNA-induced silencing complex (RISC) and exerts its regulatory function on gene expression (Fig. 2) [105, 106]. The expression of miRNAs is tissue-specific and is categorized in two groups including tumor-promoting and tumor-suppressor miRNAs [107]. Regardless of physiological function of miRNAs in cells, increasing evidence highlights their role in cancer. For instance, modulating their expression using transfection strategies can be beneficial in cancer treatment [108, 109]. In the next sections, we will provide a summary of miRNA and EZH2 interaction in different cancers and how this interaction can affect the malignancy of cancer cells.

#### Brain tumors

Brain tumors are responsible for high mortality and morbidity worldwide. Here, we cover the role of miRNAs in cancer progression and inhibition [112, 113]. Glioma is a well-known brain tumor with an upregulated EZH2 expression [114]. On the other hand, miRNA-32 is a tumor-suppressor factor undergoing down-regulation in glioma cells and tissues. Enhancing miRNA-32 expression significantly impairs proliferation and migration of glioma cells via EZH2 down-regulation [115]. The association between miRNA and EZH2 expression has been investigated in glioma [116]. MiRNA-340 is considered as a tumor-suppressor factor in which its upregulation increases anti-tumor immunity and promotes macrophage phagocytosis [117]. Furthermore, miRNA-340 can suppress cancer metastasis via down-regulating RhoA expression [118]. In glioblastoma multiforme, miRNA-340 stimulates apoptosis and cell cycle arrest while suppressing cancer cell metastasis. Furthermore, this miRNA induces autophagy and differentiation. It is thus suggested that EZH2 down-regulation by miRNA-340 is involved in anti-tumor activities [99].

The LIM-only protein 3 (LMO3) was first identified in glioma and is considered as a DNA methylation gene [119]. LMO3 has been implicated in brain tumors and can enhance cancer proliferation and tumorigenesis in neuroblastoma [120, 121]. By inhibiting EZH2, miRNA-101 diminishes promoter occupation of LMO3 by H3K27me3 and prevents its methylation, resulting in glioma suppression [122]. In fact, miRNAs target EZH2 to affect the methylation condition of a specific gene that is of importance for its activation or inhibition [123].

Therefore, proliferation and invasion of brain tumors are affected by miRNA/EZH2 axis [124]. In the tumor microenvironment, there are competitions among rapidly dividing and proliferating cancer cells, leading to hypoxia and subsequent induction of angiogenesis for promoting cancer progression [125, 126]. EZH2 can induce angiogenesis and promote cancer growth [127]. MiRNA-137 can bind to 3′-UTR of EZH2 to diminish its expression, impairing glioblastoma proliferation and angiogenesis [128]. Down-regulation of tumor-suppressor miRNAs results in EZH2 activation and subsequent induction of angiogenesis and growth of glioblastoma cells [129].

#### Thoracic tumors

##### Lung cancer

Among thoracic cancers, lung cancer is a common type and recent studies have highlighted the role of miRNAs in regulating its malignancy and aggressive behavior. Increasing evidence suggests that EZH2 upregulation is in favor of lung cancer progression [130]. On the other hand, there are miRNAs capable of regulating EZH2 expression in lung cancer. A recent experiment has shown that miRNA-92b as a tumor-suppressor role and binds to 3′-UTR of EZH2 to reduce its expression, impairing proliferation and metastasis of lung cancer cells [114]. Furthermore, miRNAs can regulate the behavior of cancer stem cells (CSCs) in lung cancer. Briefly, CSCs are responsible for cancer progression and inducing resistance to therapy. Furthermore, the presence of CSCs is associated with cancer recurrence [131, 132]. Therefore, it is of importance to delineate the relationship between miRNA/EZH2 axis and CSCs for developing novel therapeutics. It has been reported that miRNA-21 promotes the expression level of EZH2 and enhances the progression of CSCs to provide resistance to radiotherapy and chemotherapy in lung cancer cells. The apoptosis and cell cycle regulators including Cdc2, cyclin B1 and Bcl-2 are regulated by miRNA-21/EZH2 axis in CSCs. Silencing miRNA-21/EZH2 axis enhances the potential of radiotherapy and chemotherapy suppressing lung cancer by 39.2% and 69.7% [133]. The downregulation of EZH2 in lung cancer cells by miRNAs is mediated by its binding to 3′-UTR [134].

Methotrexate is a potent anti-tumor agent with a capacity of inhibiting dihydrofolate reductase to suppress cancer progression [135, 136]. This agent is extensively applied in lung cancer therapy, however, the development of drug resistance in cancer cells can limit its anti-tumor activity. To overcoming resistance, nanoparticles have been developed for targeted delivery of methotrexate with some degrees of success [137, 138]. Another tool that is commonly used to reduce the development of drug resistance is gene therapy. This requires an in-depth understanding of signaling networks that are involved in methotrexate resistance and the resulting genetic modulations in cellular responses. In A549 cells, EZH2 overexpression diminishes sensitivity to methotrexate therapy and prevents apoptosis and cell cycle arrest. For potentiating methotrexate-mediated cell death and cell cycle arrest, transfection of A549 cells with miRNA-200c results in down-regulation of EZH2 [139].

One of the mechanisms responsible for tumor metastasis is EMT. At morphological level, epithelial cells are transformed to mesenchymal cells that have high motility rate. At molecular level, a number of factors known as EMT-inducing transcription factors (EMT-TFs) such as ZEB1/2, Snail, Slug and TGF-β result in EMT stimulation via E-cadherin down-regulation, and N-cadherin and vimentin upregulation. These processes are reversible and its reversible type, known as MET can be induced. As EMT leads to metastasis and poor prognosis, its suppression is of importance in cancer therapy [140–144]. A recent experiment has shown how miRNA/EZH2 axis can modulate EMT in lung cancer cells. The expression of miRNA-124 undergoes down-regulation in lung adenocarcinoma compared to normal cells. MiRNA-124 transfection results in down-regulation of EZH2 and subsequent inhibition of EMT, impairing lung cancer migration [145].

##### Breast cancer

Breast cancer is one of the most encountered malignancies among women with miRNAs and EZH2 playing a significant role in proliferation, metastasis, and therapy response [146, 147]. This section is dedicated to discussing miRNA and EZH2 interaction in breast cancer cells. As a dynamic process, autophagy activation occurs when damaged proteins and organelles accumulate and their degradation is vital for preserving cell homeostasis [148, 149]. Increasing evidence demonstrates the role of autophagy induction in reducing proliferation of breast cancer cells [112]. As a tumor-suppressor factor, miRNA-92b stimulates autophagy and diminishes viability and growth of breast cancer cells. In this way, miRNA-92b binds to 3′-UTR of EZH2 to reduce its expression, resulting in the upregulation of light chain-3 (LC3) and SQSTM1 degradation [150]. However, it should be mentioned that autophagy possesses a pro-survival role and its activation may enhance breast cancer progression [151–153]. Thus, the dual role of autophagy in cancer should also be considered.

It is worth mentioning that miRNAs can exert a synergistic impact with anti-tumor agents in regulating EZH2 expression and affecting breast cancer progression. Venom peptides derived from predatory marine cone snails are considered as promising agents in cancer therapy. It has been reported that Conus peptides have a high stability, are resistance to proteases, and can selectivity target receptors [134, 154, 155]. A recent experiment has applied Syn-Cal14.1a, a synthetic peptide isolated from *Californiconus californicus* (Cal14.1a), in addition to miRNA-101-3p transfection to suppress breast cancer progression. This combination synergistically down-regulates EZH2 expression and inhibit proliferation and migration of breast cancer cells [156]. As more experiments are performed, more miRNAs involved in regulating EZH2 expression are revealed. MiRNA-340 is a tumor-suppressor and significantly diminishes proliferation and metastasis of breast cancer cells via regulating different molecular pathways such as inhibiting Wnt and ROCK1 [157, 158]. In vitro and in vivo experiments have shown that miRNA-340 transfection significantly inhibits tumor growth in vitro and in vivo. In this way, miRNA-340 down-regulates EZH2 expression to decrease DNMT1, H3K27me3, β-catenin and signal transducer and activator of transcription 3 (STAT3), an oncogenic transcription factor. This axis results in down-regulation of miRNA-21 and upregulation of miRNA-200a/b, which are of key importance in breast cancer therapy [159].

MiRNA-33a is another tumor-suppressor that impairs breast cancer metastasis and growth while promoting sensitivity to doxorubicin chemotherapy via inhibiting EMT, eukaryotic translation initiation factor 5A2 (eIF5A2)[160, 161]. In triple-negative breast cancer cells, miRNA-33a reduces growth and induces cell cycle arrest (G1 phase). Enhancing miRNA-33a expression is associated with EZH2 down-regulation and suppression of breast cancer progression [162]. It has been reported that miRNA/EZH2 axis can regulate the therapy response of breast cancer cells. Doxorubicin (DOX) is a well-known anti-tumor agent that participates in cancer suppression by inhibiting the activity of topoisomerase II and inducing cell cycle arrest [163, 164]. However, recent experiments have shown the development of DOX resistance and the need for using complementary strategies in suppressing resistance [165, 166]. One of the potential mechanisms that can be used is miRNA replacement therapy to increase the expression levels of tumor-suppressing miRNAs. In breast cancer cells, enhancing expression levels of miRNA-15a and miRNA-16 reduces the EZH2 expression and partially prevents DNA repair and provides DOX sensitivity [167].

#### Gastrointestinal tumors

##### Gastric cancer

Gastric cancer is a lethal disease and EZH2 upregulation is in favor of gastric carcinogenesis. As a tumor-suppressor, miRNA-26 suppresses EZH2 expression to impair gastric cancer progression. Investigation of molecular pathways demonstrates that TET, a member of DNA demethylases family, can provide sequestration of miRNA-26, paving the way for EZH2 overexpression and gastric tumorigenesis [168]. To enhance our capacity in enhancing miRNA expression, delivery methods including viral vectors have been used. A recent experiment uses lentivirus for delivery of miRNA-124 to down-regulate EZH2 expression and successfully inhibit to gastric cancer progression [169].

Gastrokine 1 (GKN1) has been isolated from gastric mucosa cells and is also expressed in autocrine/paracrine gastric mucosa [170, 171]. GKN1 is involved in the process of healing and protecting antral mucosa [172]. GKN1 can suppress gastric cancer proliferation via upregulating miRNA-185. EZH2 down-regulation, on the other hand, leads to inhibition of gastric cancer progression, and promotion of sensitivity to 5-fluorouracil chemotherapy [173].

##### Liver cancer

Liver cancer is a leading cause of cancer related mortality worldwide. MiRNA-26a is a tumor-suppressor factor capable of suppressing cancer progression via down-regulating matrix metalloproteinases (MMPs) [174]. Furthermore, miRNA-26a induces cancer cell apoptosis via E2F7 down-regulation [175]. In hepatocellular carcinoma, miRNA-26a stimulates cell growth inhibition via inhibiting EZH2 expression [176]. EZH2 can function as upstream mediator of STAT3 by providing methylation [177]. In hepatocellular carcinoma, miRNA-137 inhibits migration and metastasis via targeting EZH2/STAT3 signaling. In this way, miRNA-137 inhibits EZH2 and its downstream target STAT3 to provide E-cadherin upregulation and Snail down-regulation in favor of metastasis inhibition [178]. Similar to STAT3, Wnt/β-catenin is also a downstream pathway of EZH2 [179]. Many miRNAs affect Wnt signaling via targeting EZH2. Among them, miRNA-98 suppresses EZH2 expression to inhibit Wnt signaling, resulting in a decrease in hepatocellular carcinoma proliferation [180]. Interferon-alpha (IFN-γ) is an inflammatory factor and can induce the expression of miRNAs in cancer cells. In hepatocellular carcinoma, IFN-γ enhances the expression level of miRNA-26a that subsequently, down-regulates EZH2 to suppress metastasis [181]. Besides, miRNA/EZH2 axis can regulate the response of hepatocellular carcinoma cells to therapy. It has been reported that EMT induction can lead to cisplatin resistance [182]. MiRNA-138 reduces EZH2 expression to inhibit EMT, resulting in an increase in cisplatin sensitivity in hepatocellular carcinoma cells [183]. Overall, these studies agree with the fact that miRNA/EZH2 axis can affect proliferation, metastasis, and therapy response of hepatocellular carcinoma cells, and in this way, different molecular pathways such as Wnt, STAT3 and EMT are affected.

##### Colon cancer

Like other kinds of gastrointestinal tumors, EZH2 overexpression enhances colon cancer progression and its inhibition by anti-tumor agents such as salinomycin is associated with activation of death receptors [184]. MiRNA and EZH2 interaction is a determining factor for colon cancer progression. It has been reported that colon cancer metastasis is impaired following miRNA-101 overexpression and is attributed to EZH2 down-regulation and subsequent inhibition of cancer invasion [185]. It is suggested that the inhibitory impact of miRNA-101 on colon cancer metastasis is due to EMT inhibition. Overexpression of miRNA-101 inhibits EZH2 signaling post-transcriptional to suppress EMT, leading to colon cancer metastasis impairment [186].

#### Reproductive tumors

##### Prostate cancer

Prostate cancer is a common tumor among men and EZH2 has been shown to play a role in its malignancy affecting growth and senescence [187]. Furthermore, EZH2 can enhance prostate cancer metastasis via Twist upregulation and increasing N-cadherin levels [188]. The expression of miRNA-605-3p as a tumor-suppressor factor is down-regulated in prostate cancer cells, which increases the expression of EZH2 to disrupt prostate cancer growth and metastasis. These results have been shown in both in vitro and in vivo [189]. It is noteworthy that miRNAs can indirectly affect EZH2 via targeting other upstream mediators. Hypoxia diminishes miRNA-137 expression to promote migration of prostate cancer cells via EMT induction [190]. A recent experiment has shown that miRNA-137-3p is down-regulated in prostate cancer cells, and its low expression is associated with tumor stage. MiRNA-137-3p down-regulates EZH2 expression via c-Jun N-terminal kinase 3 (JNK3) inhibition which significantly suppresses prostate cancer proliferation and metastasis, while stimulating apoptosis [191].

The therapeutic targeting of miRNA/EZH2 axis has been shown to suppress prostate cancer progression. The Aristeromycin (a derivative of 3-deazaneplanocin A (DZNeP) has been effective at upregulating the expression level of miRNA-26a. In turn, miRNA-26a binds to 3′-UTR of EZH2 to reduce its expression and impair prostate cancer proliferation [192]. There is a close relationship between miRNAs and androgen receptor (AR) signaling in enhancing prostate cancer progression. Briefly, AR signaling plays a key role in prostate cancer development and progression, and androgen deprivation therapy (ADT) is used to suppress prostate cancer progression [193]. As a tumor-promoting factor, AR signaling enhances EZH2 expression to inhibit apoptosis and provide enzalutamide resistance. Intracellular delivery of miRNA-124 reduces AR splice variants and suppresses EZH2 as a downstream target to prevent prostate cancer progression [194].

#### Gynecological tumors

##### Ovarian cancer

**Fig. 3:**
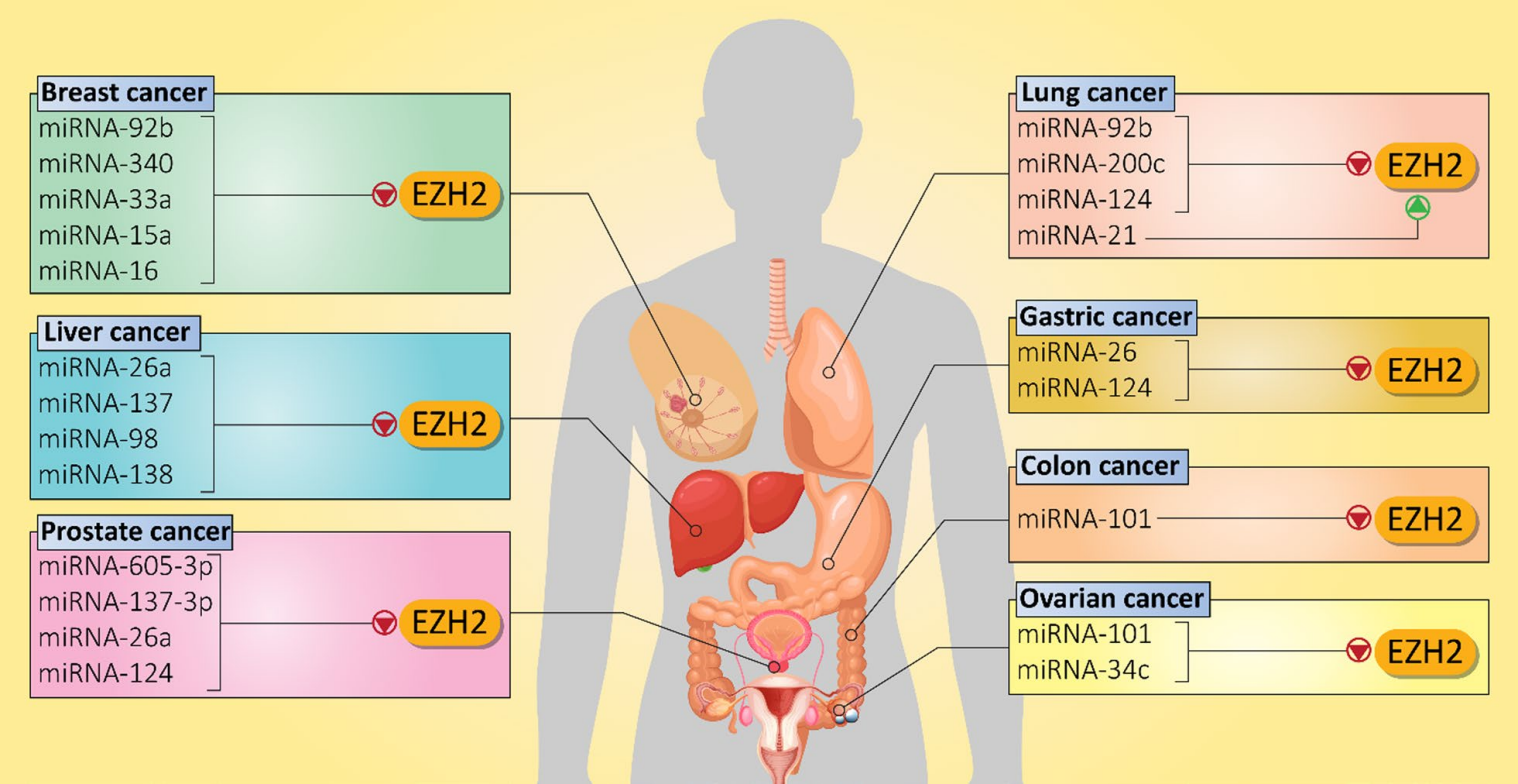
MiRNAs regulating EZH2 in different cancers

Ovarian cancer is a common gynecologic cancer with a high global mortality rate. Both miRNAs and EZH2 demonstrate abnormal expressions in ovarian cancer [195–198]. Indeed, the miRNA/EZH2 interaction determines progression and chemoresistance of ovarian cancer cells. MiRNA-101 is observed at low levels in ovarian cancer. Enhancing miRNA-101 expression is associated with EZH2 down-regulation and a subsequent decrease in proliferation and migration of ovarian cancer cells. Furthermore, as aggressive behavior of these malignant cells decreases, their sensitivity to cisplatin enhances [199]. It has been reported that EZH2 increases ovarian cancer growth and promotes cell cycle progression through transcriptional activation of CDKN1C. It is noteworthy that miRNA-34c inhibits EZH2 in favor of reducing ovarian cancer proliferation (Table 3) [200]. A few studies have examined the role of miRNA/EZH2 interaction in ovarian cancer and more experiments are needed to delineate the mechanisms of action. Furthermore, studies on regulation of miRNA/EZH2 axis by lncRNAs are discussed below. Figure 3 shows regulation of EZH2 signaling by miRNAs in different cancers.

**Table 3:** MiRNAs as potential upstream mediators of EZH2 signaling in cancers

MiRNA	Signaling network	Cancer type	In vitro*/*in vivo	Cell line/Animal model	Remarks	References
MiRNA-876-3p	SPRR3/EZH2	Non-small cell lung cancer	In vitro In vivo	H1299, PC9, HCC827 and A549 cells NOS/SCID mice	Exerting an anti-tumor function Inhibiting SPRR3/EZH2 axis Apoptosis induction Disrupting metastasis	[201]
MiRNA-21	–	Lung cancer	In vitro	A549 cell line	MiRNA-21 promotes proliferation and therapy response of cancer cells Silencing miRNA-21 and its downstream target EZH2 enhance therapy sensitivity	[133]
MiRNA-200c	–	Lung cancer	In vitro	A549 cells	Inhibiting migration and invasion of cancer cells via enhancing E-cadherin levels and decreasing EZH2 expression	[139]
MiRNA-124	–	Pancreatic cancer	In vitro	AsPC-1, PANC1, BxPC-3 and SW1990 cells	Impairing metastasis and proliferation of cancer cells Delivery to tumor cells via exosomes EZH2 overexpression disrupts anti-tumor activity of miRNA-124	[202]
MiRNA-137	EZH2/LSD1	Endometrial cancer	In vitro In vivo	AN3CA, HEC1A, KLE, RL-95-2 cells Xenografts	Suppressing proliferation of cancer cells Inhibiting EZH2 and LSD1 expression levels	[203]
MiRNA-494	MYC/EZH2	Burkitt lymphoma	In vitro	BL cell lines	The MYC can increase EZH2 expression in maintaining malignancy of lymphoma cells MiRNA-494 inhibits MYC/EZH2 axis	[204]
MiRNA-26a	–	Bladder cancer	In vitro	EJ cells	Apoptosis induction Decreasing proliferation of cancer cells MiRNA-26a inhibits EZH2 signaling	[205]
MiRNA-98	EZH2/Wnt/β-catenin	Hepatocellular carcinoma	In vitro	HCCLM3, HepG2, SMMC7721, Hep3 B cell lines	Binding to 3′-UTR of EZH2 and reducing its expression Inactivating Wnt signaling Suppressing cancer growth	[180]
MiRNA-378a-3p MiRNA-378d	EZH2/STAT3	Breast cancer	In vitro In vivo	CAL51, MDA-MB-231 and MCF-7 cells Xenografts	Upregulation of EZH2 and subsequent induction of STAT3 signaling in increasing chemoresistance feature and stemness of breast cancer cells	[206]
MiRNA-124	EZH2/STAT3	Cholangiosarcoma	In vitro In vivo	HuCCT1, KMBC, and MZChA1 cells Mouse xenograft model	MiRNA-124 inhibits EZH2 and its downstream target STAT3 Inducing autophagy-related cell death via ATG5 upregulation Reducing miRNA-124 expression enhances disease progression	[207]
MiRNA-26a	EZH2	Skin cancer	In vitro	HaCaT cells	Reducing EZH2 expression to mediate UV-induced apoptosis Using EZH2 inhibitors aggravates apoptosis	[208]

### EZH2 as upstream mediator of miRNA expression

The previous section highlighted the role of miRNAs as upstream mediators of EZH2 in cancer regulation. It is noteworthy that there are studies demonstrating that EZH2 can regulate miRNAs and result in cancer progression. Glycolysis or Warburg effect is responsible for increased glucose uptake and higher metabolism ensures rapid proliferation of cancer cells. With respect to the role of glycolysis in cancer progression, various inhibitory therapeutic agents have been developed [16, 209]. EZH2 upregulation in prostate cancer cells results in the induction of hexokinase-2 (HK2) to increase glycolysis. MiRNA-181b as a tumor-suppressor factor binds to 3′-UTR of HK2 to inhibit glycolysis and progression of prostate cancer cells. However, transcriptional knockdown of EZH2 activates HK2-mediated glycolysis [210]. In addition, miRNA regulation by EZH2 can affects cell cycle progression. It has been reported that miRNA-200c binds to 3′-UTR of E2F3 to reduce its expression, inducing cell cycle arrest. In turn, EZH2 as an upstream mediator, down-regulates miRNA-200c expression to induce E2F3-mediated cell cycle progression in prostate cancer [211].

EZH2 can also interact with other tumor-promoting factors such as YY1 to down-regulate miRNA expression. YY1 is a zinc finger transcriptional factor that exerts a variety of biological activities including cell growth, differentiation, apoptosis, embryonic development, and carcinogenesis [212–214]. YY1 recruits EZH2 in prostate cancer cells to down-regulate miRNA-146a expression. Silencing YY1 enhances miRNA-146a expression to induce apoptosis in cancer cells [215]. SOX4 is considered as a tumor-promoting factor in ovarian cancer and its upregulation by lncRNA FEZF1-AS1 results in cancer inhibition [216]. It is noteworthy that SOX4 can function as an upstream mediator of EZH2 to induce H3K27me3. This axis leads to down-regulation of miRNA-212 and 132 and activating EMT for ovarian cancer metastasis [217].

It has been reported that EZH2 can recruit factors responsible for DNA methylation such as DNA methyltransferases enzymes (DNMTs) that induce hypermethylation of CpG islands [218]. In cervical cancer cells, EZH2 recruits DNMT1 to silence miRNA-484 via triggering methylation of its promoter. Subsequently, activation of membrane-bound matrix metalloproteinase (MMP14) and the hepatocyte nuclear factor 1A (HNF1A) drive cancer metastasis and invasion [219].

The miRNA-125a is a tumor-suppressor and its upregulation by propofol results in reduced growth and invasion of gastric cancer cells [220]. MiRNA-125a can be considered as a diagnostic factor for gastric cancer [221], enhancing expression level of breast cancer metastasis suppressor 1 (BRMS1) to inhibit metastasis and migration of gastric cancer cells. EZH2 as an upstream mediator down-regulates miRNA-125a and BRMS1 to enhance gastric cancer metastasis [222].

**Fig. 4:**
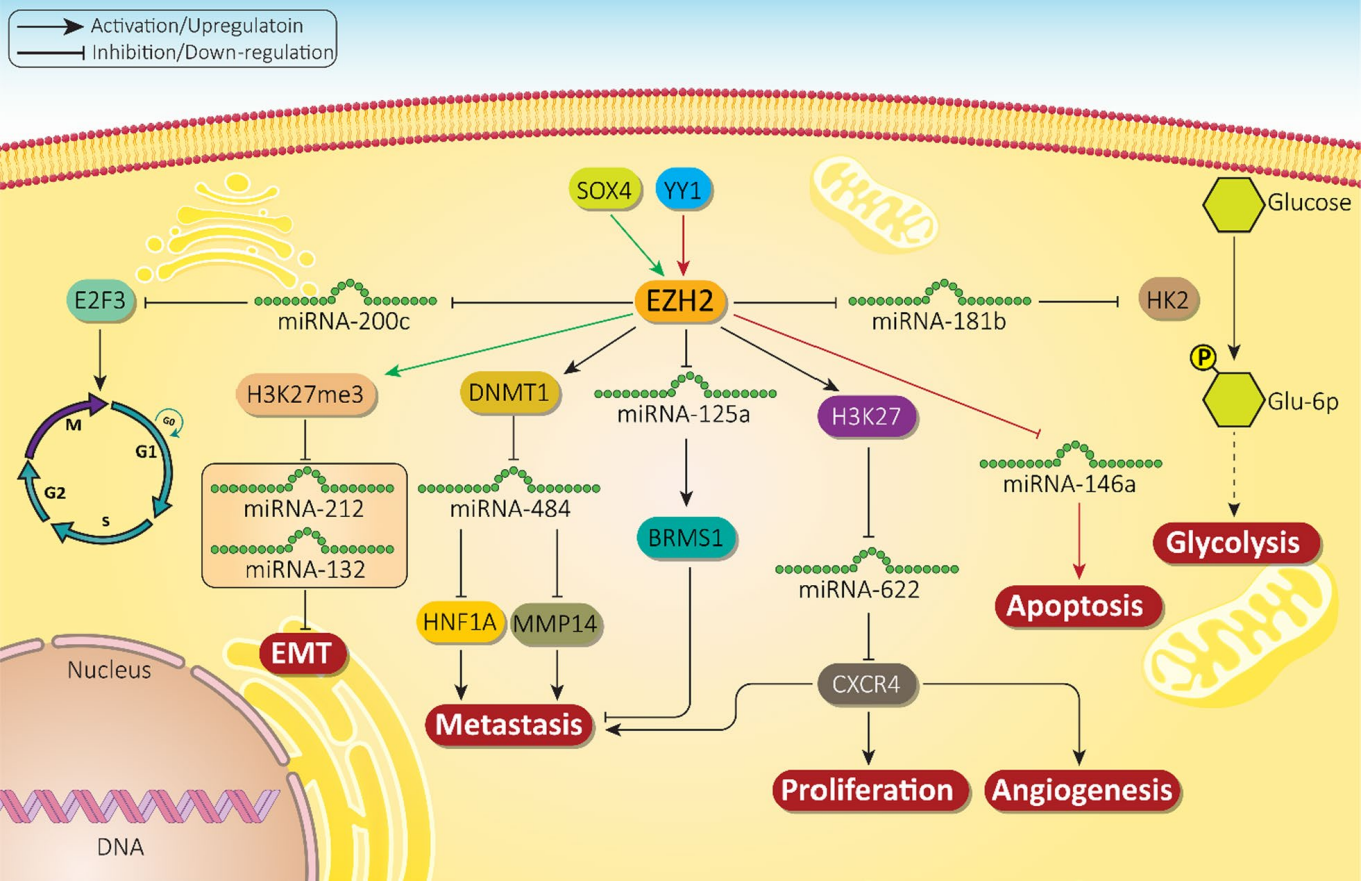
EZH2 as upstream mediator of miRNA expression in cancers. Due to transcriptional role of EZH2, this pathway can target miRNAs in affecting proliferation and invasion of cancer cells that subsequently, determine response of cancer cells to therapy

The CXC chemokine receptor 4 (CXCR4) is a receptor for chemokine ligand 12 (CXCL12) and regulates various biological events in cells including angiogenesis, EMT, dissemination, migration, and cell stemness [223, 224]. By binding to 3′-UTR of CXCR4, miRNA-622 down-regulates its expression. EZH2 activates trimethylation of miRNA-622 promoter at H3K27 to diminish its expression, leading to CXCR4 upregulation and increased progression of hepatocellular carcinoma [225]. Overall, owing to the trimethylation activity of EZH2 at H3K27 site, the expression of miRNAs can be regulated through promoter transcriptional suppression [226–236]. Figure 4 depicts role of EZH2 in regulation of miRNAs in different cancers.

### Synthetic short non-coding RNAs

#### SiRNAs

The small interfering RNA (siRNA) is a kind of RNA interference (RNAi) consisting of double-stranded RNA and has a homologous sequence to target gene [237, 238]. SiRNAs can down-regulate the expression of target genes (silencing), a mechanism that begins in the cytoplasm with the aid of Dicer enzymes that cleaves double-stranded RAN to produce shorter RNA molecules, known as siRNA with length of 21–25 nucleotides. For this purpose, the stranded guide is loaded in RISC and after matching with a target mRNA, cleaves it and results in gene silencing. As gene deregulation is a common phenomenon in different diseases, especially cancer, a wide variety of experiments have applied siRNA for gene silencing to suppress cancer progression [239]. Despite significant progress in gene identification and further application of siRNA, the use of siRNA in clinical course is limited. There are several reasons for limitations in the clinical application of siRNA. The first reason is that siRNA should overcome physiological and cellular barriers that prevent entrance of siRNA into cytoplasm of cells. Furthermore, as RNAses are present in different locations in cells and tissues, they can degrade siRNAs and inhibit their potential in gene silencing [240]. For overcoming the aforementioned challenges, delivery systems have been designed to improve the potential of siRNA in gene silencing [241]. In this section, we discuss the role of siRNAs and delivery systems to silence EZH2 and suppress cancer malignancy.

With respect to the tumor-promoting role of EZH2, its inhibition should significantly diminish the survival of cancer cells. In this way, siRNA-EZH2 reduces the proliferation of bladder cancer cells by 37.9% and migration by 67% [242]. Furthermore, EZH2 upregulation is in favor of chemoresistance and reducing the sensitivity of cancer cells to chemotherapy [206, 243]. EZH2 down-regulation by siRNA is associated with apoptosis induction (caspase 3/8 activation) and cell cycle arrest (G0/G1 phase). Besides, EZH2 silencing by siRNA results in the downregulation of tumor-promoting factors including cyclin D1 and multidrug resistance 1 (MDR1) at protein and mRNA levels. On the other hand, EZH2 silencing upregulates tumor-suppressor factors such as p15, p21, p27 and miRNA-218 that can in turn suppress the progression of lung and gastric cancers and enhance their sensitivity to cisplatin chemotherapy [244]. However, the efficacy of siRNAs needs to be improved to gain better results in gene silencing. Polymeric nanoparticles can promote the intracellular accumulation of siRNA-EZH2 up to 98%. Due to their low size (35.6 nm), nanoparticles demonstrate high stability as confirmed by their zeta potential of 36.7 mV. Both in vitro and in vivo experiments reveal the role of siRNA-EZH2-loaded polymeric nanoparticles in gene silencing and subsequent apoptosis induction in cancer cells [245]. Co-delivery of siRNAs with other chemotherapeutic agents can be provided via nanocarriers. This leads to their synergistic impact and improvement in cancer elimination. Furthermore, surface modification of nanoparticles, for instance by RGD peptide, can enhance the selectivity of nanoparticles towards cancer cells and in EZH2 silencing [246]. Compared to siRNA-EZH2 or cisplatin alone, cisplatin- and siRNA-EZH2-loaded nanoparticles induce more toxicity against cancer cells, showing a synergistic impact capable of reversing chemoresistance [247]. As siRNA-EZH2 nanoparticles are administered systemically for cancer suppression, a special attention should be directed towards their biocompatibility and low toxicity towards normal cells [248]. Future experiments need to focus on developing green-synthesized nanocarriers for siRNA-EZH2 delivery and examine downstream targets of EZH2 in normal cells (Table 4).

**Table 4:** The role of SiRNAs and nanoscale delivery systems in regulating EZH2 in cancer therapy

Cancer type	Nanocarrier	Co-delivery	In vitro*/*in vivo	Cell line/Animal model	Remarks	References
Bladder cancer	–	–	In vitro	T24 cells	EZH2 down-regulation by siRNA Reducing growth up to 37.9% Decreasing metastasis up to 67%	[242]
Non-small cell lung cancer Gastric cancer	–	–	In vitro	AGS and A549 cells	Inducing cell cycle arrest at G0/G1 phase after siRNA-EZH2 application Apoptosis stimulation Caspase-3/8 activation Down-regulation of cyclin D1 and MDR1 Upregulation of p15, p21, p27 and miRNA-218 as tumor-suppressor factors	[244]
Non-small cell lung cancer	Multifunctional nanoparticles	SiRNA-EZH2 Etoposide	In vitro In vivo	A549 cells Orthotopic lung cancer model	Reducing mRNA and protein levels of EZH2 Decreasing proliferation and invasion of cancer cells Selective targeting tumor cells via RGD modification Synergistic impact	[246]
Glioma	Polymeric nanoparticles	–	In vitro In vivo	U87 cells Tumor-bearing mice	High transfection efficiency (up to 98%) Zeta potential of 36.7 demonstrates high stability Particle size of 35.6 nm Providing gene silencing and suppressing cancer progression	[176]
Ovarian cancer	Iron nanoparticles	Platinum siRNA	In vitro In vivo	A2780 cells Tumor-bearing mice	Synergistic impact for overcoming drug resistance Cancer elimination Apoptosis induction	[247]

#### ShRNA

The short-hairpin RNA (shRNA) is like siRNA in gene silencing. ShRNAs are 19–20 nucleotides in length with a short hairpin loop of 4–11 nucleic acids. ShRNA is transcribed from DNA with the aid of RNA polymerase II and III. Pre-shRNAs translocate to cytoplasm by Exportin-5, where they form complexes with Dicer enzymes and are loaded in RISC for cleavage [249]. To date, few experiments have investigated the role of shRNAs in EZH2 silencing and suppressing cancer progression. These studies have applied delivery systems including nanocarriers and biological carriers for shRNA silencing of EZH2. Polymeric nanoparticles and adenoviruses for shRNA-EZH2 delivery have high transfection efficiency, great potential in gene silencing and lead to effective suppression of proliferation and invasion of prostate cancer cells, reduce expression of EZH2 and its downstream targets including Ki67 and CCND1 [250, 251]. Although no absolute conclusions can be made from these experiments, they demonstrate the efficiency of shRNA in EZH2 silencing and further studies can provide a comparative investigation of the role of shRNA and siRNA in EZH2 silencing and their potential.

### LncRNAs and EZH2 signaling

**Fig. 5:**
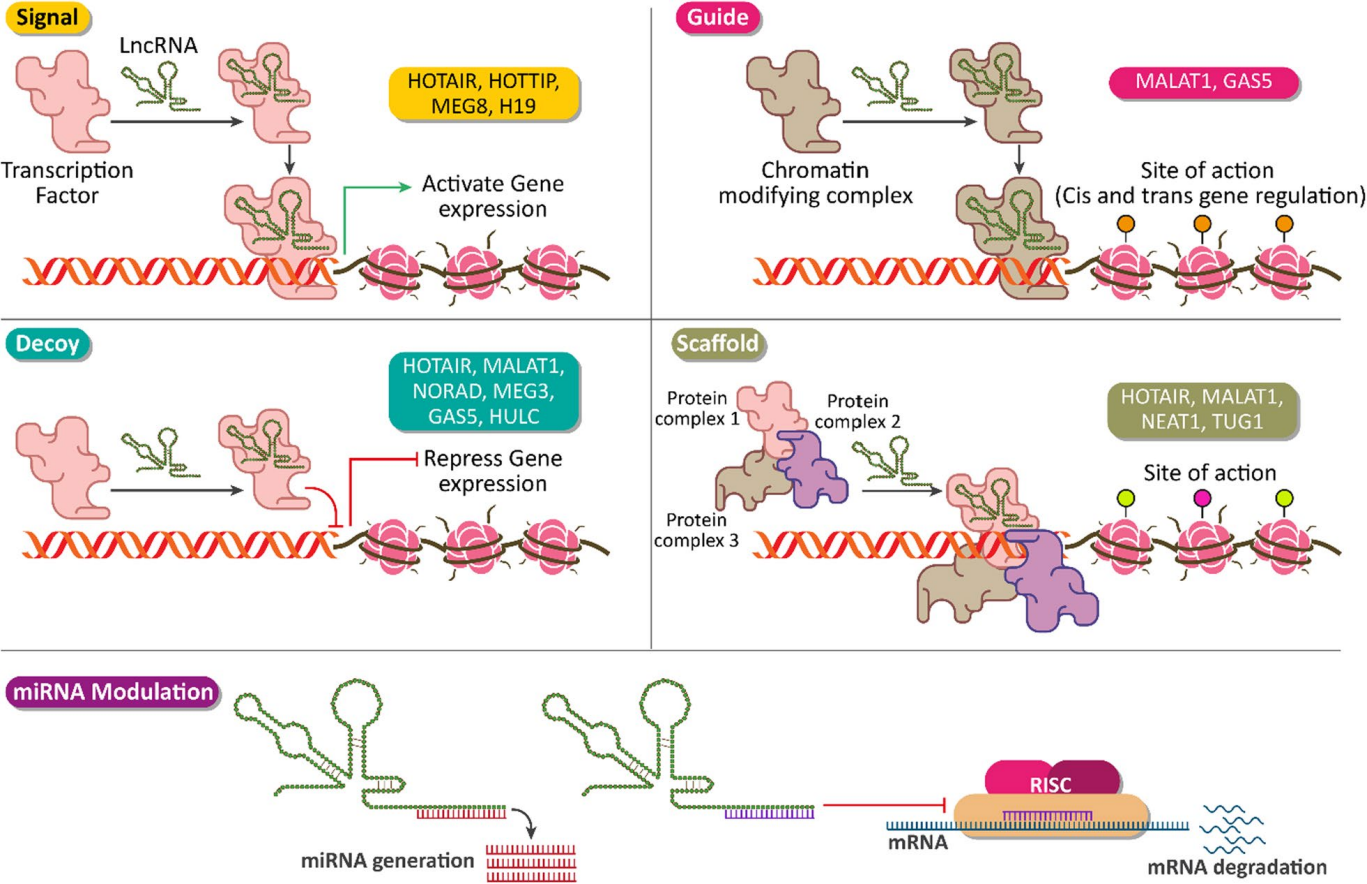
The lncRNA function in cells [268]

Long non-coding RNAs (lncRNAs) are important members of ncRNAs with a length more than 200 nucleotides and do not encode any proteins (Fig. 5) [230, 252]. As most part of genome is formed by ncRNA encoded by junk DNA, they are not transcribed to proteins [253, 254]. However, this concept was changed after the discovery of potential roles of lncRNAs in biological events [255]. LncRNAs can affect gene expression at various levels including chromatin, transcriptional and post-transcriptional levels [256]. Increasing evidence has revealed the role of lncRNAs in differentiation, cell cycle, and stem cell pluripotency [257–260]. It is suggested that lncRNAs are important in cancer biology and can affect proliferation, metastasis, and therapy response of cancer cells [10, 261–264]. By affecting other ncRNAs or proteins, lncRNAs can function as signals, guides, decoys or scaffolds to change cellular functions [265, 266]. Similar to miRNAs, lncRNAs can have both tumor-suppressing and tumor-promoting functions as suggested by been different experiments [263]. Single-cell sequencing has shown that lncRNAs have heterogeneous roles in individual cancer cells [267]. Therefore, it is of interest to explore the role of lncRNAs in different types of cancer. In the next section, we examine the role of lncRNAs in regulating EZH2 expression in various cancers.

#### LncRNAs inducing EZH2

The lncRNA small nucleolar RNA host gene 1 (SNHG1) is a tumor-promoting factor and recent studies have revealed its role in cancer progression. This lncRNA induces phosphoinositide 3-kinase (PI3K)/protein kinase-B (Akt) to enhance bladder cancer proliferation and metastasis [269]. Furthermore, SNHG1 can regulate miRNA expression to provide chemoresistance [270]. In colorectal cancer cells and tissues, SNHG1 is increased in expression and is associated with decreased patient survival. Investigation of molecular pathways reveals down-regulation of miRNA-154-5p by SNHG1 via sponging and EZH2 induction which increases colorectal cancer progression [271]. Another study reveals the role of SNHG1 in bladder cancer progression via regulating EZH2. In this way, SNHG1 enhances EZH2 expression in the nucleus to down-regulate CDH1, resulting in a decrease in E-cadherin levels and promoting metastasis and migration of bladder cancer cells [272].

LncRNA SNHG6 is another factor with a potential role in cancer. SNHG6 upregulation is associated with poor prognosis of patients with colorectal cancer [273]. SNHG6 can also enhance radio-resistance of cervical cancer cells [274]. In colorectal cancer cells, SNHG6 overexpression occurs due to SP1 stimulation and DNA copy number gains. SNHG6 enhances both migration and proliferation of cancer cells. Mechanistically, SNHG6 down-regulates the expression levels of miRNA-214 and miRNA-26a/b via sponging to enhance EZH2 expression, leading to colorectal cancer progression [275]. Interestingly, most of the studies have focused on the role of miRNA sponging by lncRNAs and subsequent induction of EZH2 [276]. However, lncRNAs can also interact with miRNAs to regulate EZH2. For instance, lncRNA plasmacytoma variant translocation I (PVT1) interacts with EZH2 to down-regulate miRNA-214 expression, leading to an increase in proliferation and metastasis of ovarian cancer cells [277]. Furthermore, lncRNA LINC00114 can induce EZH2/DNMT1 to reduce miRNA-133b expression and promote colorectal cancer progression [278]. Such interactions are in favor of cancer metastasis. EZH2 upregulation can promote cancer metastasis via EMT induction [279]. The lncRNA taurine upregulated gene 1 (TUG1) cooperates with EZH2 to down-regulate miRNA-382 and aid in sponging, leading to EMT induction and pancreatic cancer invasion [280].

Therapeutic targeting of lncRNAs has been tested in cancer therapy. Curcumin is a phytochemical isolated from root and rhizome of *Curcuma longa* and reveals anti-tumor activities via regulating the expression level of ncRNAs [281–285]. It has been reported that curcumin administration can promote sensitivity of pancreatic cancer cells to gemcitabine via regulating the expression level of PVT1 and its interaction with EZH2. In this way, curcumin suppresses the expression of EZH2 as a subunit of PRC2 and its related lncRNA PVT1 to prevent pancreatic cancer progression and increase gemcitabine sensitivity [286]. The lncRNA metastasis-associated lung adenocarcinoma transcript 1 (MALAT1) can promote gastric cancer progression via reducing miRNA-124-3p expression and subsequent induction of EZH2 signaling. It has been reported that exposing gastric cancer cells to hydrogen is associated with down-regulation of MALAT1, and subsequent upregulation of miRNA-124-3p as a tumor-suppressor, resulting in EZH2 inhibition and suppression of proliferation and invasion [287].

Exosomes are minute structures with sizes of 40–150 nm that are secreted by most cells [288]. The formation process of exosomes includes plasma membrane budding inward to produce early endosomes. Subsequently, late endosomes, known as multivesicular bodies (MVBs) form and are secreted to extracellular space [289–292]. It is noteworthy that exosomes can function as carriers within cells to contain proteins, lipids, and nucleic acids. Exosomes can also transfer lncRNAs in different cancers and depending on the function of lncRNAs exert cancer progression or inhibition effects [293, 294]. For instance, lncRNA UFC1 is suggested to promote lung cancer progression. UFC1 is upregulated in lung cancer tissues, serum, and serum exosomes of patients with non-small cell lung cancer and is suggested to mediate their proliferation and invasion. Silencing UFC1 is associated with cell cycle arrest and apoptosis induction. Investigation of molecular pathways demonstrates that lncRNA UFC1 binds to EZH2 to increase its accumulation at promoter of phosphatase and tensin homolog (PTEN). This leads to the trimethylation of H3K27 which subsequently down-regulates PTEN expression and promotes lung cancer progression. This suggests the key role of exosomes in facilitating the transfer of UFC1 to non-small cell lung cancer cells [295].

It was reported that miRNA/EZH2 axis affects EMT and metastasis of cancer cells. Importantly, lncRNAs can affects EMT via EZH2 regulation. The lncRNA H19 is a tumor-promoting factor capable of enhancing cancer proliferation and invasion via p53 down-regulation and subsequent induction of TNFAIP8 [274]. Silencing H19 is in favor of apoptosis induction and impairing breast cancer proliferation [296]. On the other hand, lncRNA H19 induces STAT3 expression to upregulate EZH2, leading to EMT induction in esophageal cancer cells [297]. A similar pathway by lncRNA NRON occurs to increase bladder cancer metastasis. Mechanistically, NRON stimulates EZH2 signaling to provide EMT induction and bladder cancer migration [298]. Overall, these studies agree with the regulatory role of EZH2/EMT axis by lncRNAs in different cancers. Interestingly, there is a close relationship between EMT and chemoresistance. It has been reported that EMT induction can provide paclitaxel (PTX) resistance in cancer cells [196]. The lncRNA PVT1 interacts with EZH2 to recruit it at promoter region of miRNA-195 and reduce its expression, resulting in PTX-mediated EMT. Silencing PVT1 is associated with increased sensitivity of cervical cancer cells to chemotherapy [299]. Overall, experiments agree with the fact that lncRNAs can induce cancer progression via inducing EZH2 expression. In most cases, lncRNAs stimulate EZH2 via miRNA sponging. Furthermore, it has been reported that lncRNAs recruit EZH2 to the promoter of miRNAs and other factors to reduce their expression, resulting in cancer malignancy and progression. Finally, proliferation and metastasis of cancer cells are regulated by lncRNA/EZh2 axis [296, 300–311].

#### LncRNAs inhibiting EZH2

It is noteworthy that most of studies have focused on revealing the role of tumor-promoting lncRNAs in cancer progression via EZH2 regulation. However, there are evidence showing that lncRNAs can also inhibit EZH2 signaling to function as tumor-suppressor factors. In this section, we will discuss lncRNAs capable of inhibiting EZH2. Previously, it was mentioned that chemoresistance occurs in cancer cells due to activation of tumor-promoting factors. It seems that lncRNA/EZH2 axis can also regulate the response of cancer cells to radiotherapy. The lncRNA MAGI2-AS3 is down-regulated in esophageal cancer cells. Enhancing MAGI2-AS3 expression is associated with recruitment of EZH2 to induce H3K27me3, leading to HOXB7 down-regulation and increasing radio-sensitivity [312]. This study demonstrates the tumor-suppressing role of EZH2 in cancer. Due to the methyltransferase role of EZH2, its recruitment is key in regulating the expression of other genes. For instance, as a tumor-suppressor, lncRNA MEG3 binds to EZH2 resulting in H3K27-mediated trimethylation of Engrailed-2 (EN-2) to suppress prostate cancer progression [313]. Therefore, EZH2 can also function as transcriptional regulators.

Although previous experiments demonstrate the anti-tumor activity of EZH2 in cancers via reducing expression levels of downstream targets, it appears that its down regulation by lncRNAs can reduce cancer progression. The lncRNA ANCR plays a dual role as tumor-suppressor or tumor-promoter, demonstrating that more experiments are required to reveal the role of lncs in cancer [314–317]. Specifically, ANCR exerts anti-tumor activity in breast cancer via regulating EZH2 expression. In this way, ANCR increases CDK1 and EZH2 interaction that is vital for enhancing EZH2 phosphorylation at threonine-345 and threonine-487. This phosphorylation provides the conditions for EZH2 ubiquitination and its subsequent degradation, resulting in a significant decrease in metastasis and migration of breast cancer cells [318].

Glycogen synthase kinase-3beta (GSK-3β) is considered as a driver of cancer progression and is a downstream target of PI3K/Akt axis in increasing pancreatic cancer growth and migration [319, 320]. Increased phosphorylation of GSK-3β is in favor of chemoresistance induction [321]. On the other hand, lncRNA brain-derived neurotrophic factor antisense (BDNF-AS) is suggested to impair the malignancy of colorectal cancer cells. BDNF-AS recruits EZH2 to down-regulate GSK-3β [322] and silence the tumor-promoting gene GSK-3β.

LncRNAs can affect stem cell regulation and progression of cancer cells [323]. On the other hand, CSCs play a significant role in mediating therapy resistance [324, 325]. Therefore, it is of importance to reveal how lncRNA and EZH2 interactions affect CSC’s behavior. The lncRNA STXBP5-AS1 significantly diminishes proliferation and survival of cancer cells and is associated with favorable prognosis of pancreatic cancer. This lncRNA reduces stemness of pancreatic cancer via suppressing stem cell-like features. Mechanistically, this anti-tumor activity is mediated via recruiting EZH2, suggesting its role in tumor suppression [326]. Suppressing the expression of such lncRNAs increases self-renewal capacity of CSCs in favor of cancer progression [327]. Although a few studies have examined tumor-suppressor lncRNAs, more experiments are needed to reveal lncRNA and EZH2 interaction in cancer.

#### EZH2 in lncRNA regulation

**Fig. 6:**
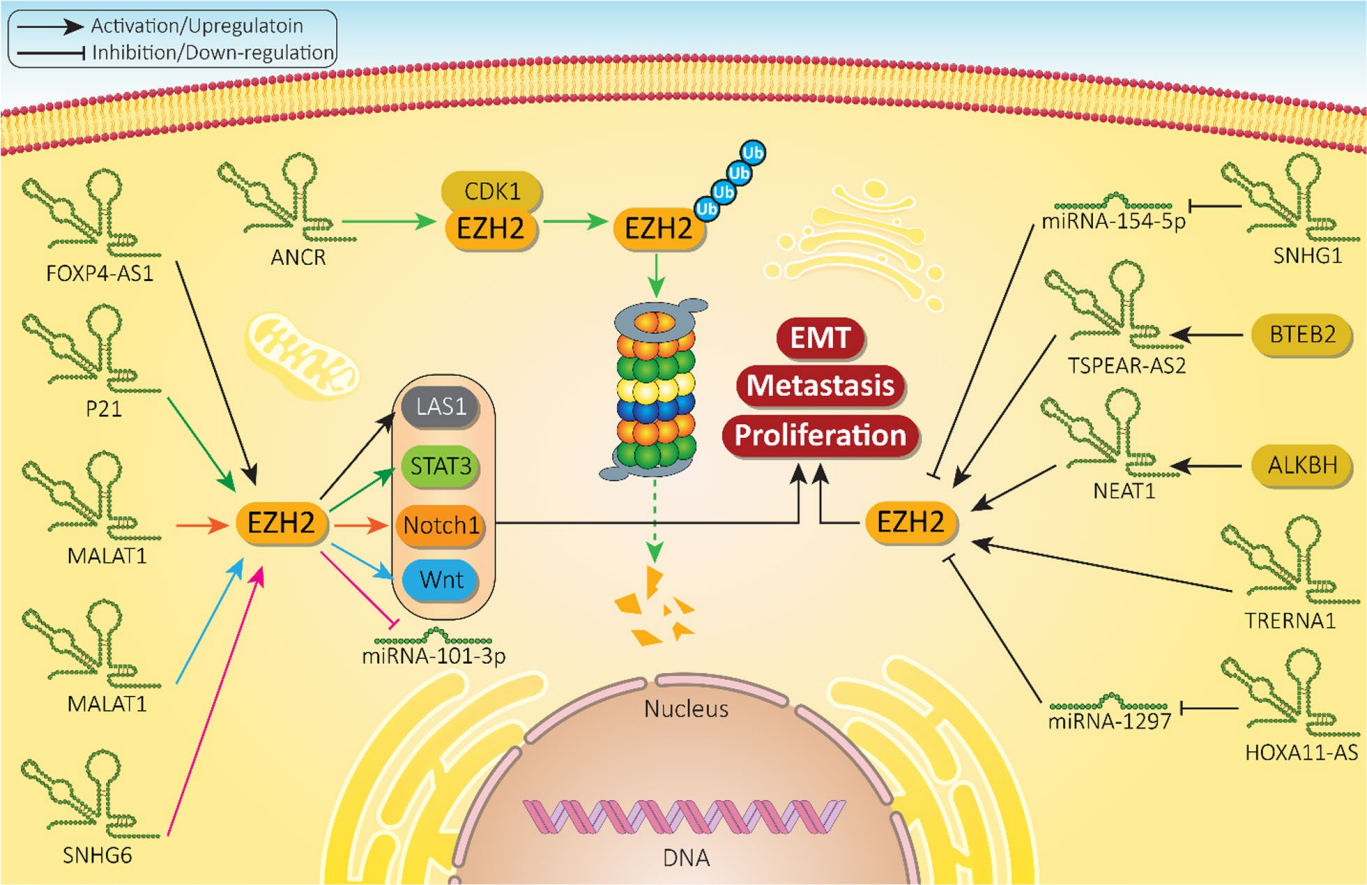
Regulation of EZH2 by lncRNAs. LncRNAs not only affect EZH2, but also its downstream targets including LAS1, STAT3, Notch1 and Wnt are affected, leading to a significant change in EMT, metastasis and growth of cancer cells

Like miRNAs, it has been reported that lncRNA expression can be regulated by EZH2 in cancer cells. Although a few studies have investigated this interaction, future experiments will shed more light on their relationship. A recent experiment has shown that lncRNA SVUGP2 is downregulated by EZH2 in lung cancer cells. Increasing expression level of lncRNA SVUGP2 is associated with impairing proliferation and invasion of lung cancer cells. Mechanistically, EZH2 down-regulates SVUGP2 to induce Wnt/β-catenin signaling to exert its tumorigenesis role (Fig. 6 and Table 5) [301].

**Table 5:** The role of lncRNAs in regulating EZH2 in different cancers

LncRNA	Signaling network	Cancer type	Remarks	References
NEAT1	ALKBH/NEAT1/EZH2	Gastric cancer	Overexpression of ALKBH in cancer cells and tissues Demethylation of NEAT1 for its activation Upregulation of EZH2 and enhancing cancer progression	[328]
TSPEAR-AS2	BTEB2/TSPEAR-AS2/EZH2/GJA1	Gastric cancer	Upregulation of lncRNA by BTEB2 Driving cancer progression via enhancing EZH2 expression and down-regulating GJA1	[329]
TRERNA1	EZH2/CDH1	Gastric cancer	Enhancing cancer metastasis via upregulating EZH2 and subsequent inhibition of CDH1, leading to EMT induction	[330]
SNHG6	EZH2/miRNA-101-3p/ZEB1	Gastric cancer	Transcriptional inhibition via recruiting EZH2 Inducing EMT via miRNA-101-3p down-regulation and subsequent stimulation of ZEB1 expression Increasing cancer metastasis	[331]
LINC00460	EZH2/LSD1/CCNG2	Gastric cancer	Overexpression of lncRNA in cancerous tissues compared to normal tissues Inducing EZH2/LSD1 axis to down-regulate CCNG2 expression Enhancing cancer progression	[332]
HOXA11-AS	MiRNA-1297/EZH2	Gastric cancer	Reducing miRNA-1297 expression via sponging Inducing EZH2 expression and its complex formation with histone demethylase LSD1 or DNMT1 Increasing cancer cell growth	[333]
FOXP4-AS1	EZH2/LSD1	Gastric cancer	Facilitating proliferation and metastasis of cancer cells Inducing EZH22/LAS1 axis	[334]
P21	EZH2/STAT3	Prostate cancer	Increased transcription of lncRNA p21 by enzalutamide through activating androgen signaling Activating non-histone methyltransferase activity of EZH2 STAT3 methylation and subsequent induction of NED	[335]
MALAT1	–	Prostate cancer	Recruitment of EZH2 and enhancing its tumorigenesis activity	[336]
MALAT1	EZH2/Notch1	Esophageal cancer	Inducing EZH2/Notch1 axis to promote metastasis via EMT induction	[337]
HERES	EZH2/Wnt	Esophageal squamous cell carcinoma	Interaction of HERES with EZH2 through G-quadruple structure-like motif Activating Wnt signaling Enhancing growth, migration and colony formation capacity of cancer cells	[338]
CASC9	EZH2/PDCD4	Esophageal squamous cell carcinoma	Association with poor survival of cancer patients Increasing cancer growth Enriching EZH2 Reducing PDCD4 expression after binding of EZH2 to its promoter	[339]
HOXA-AS2	EZH2/LSD1	Pancreatic cancer	Increasing cancer growth and survival Apoptosis inhibition Enhancing cell cycle progression Inducing EZH2/LSD1 axis	[340]
AGAP2-AS1	RREB1/AGAP-AS1/EZH2	Pancreatic cancer	Overexpression of lncRNA by RREB1 Transcriptional repression of ANKRD1 and ANGPTL4 to activate EZH2 signaling Increasing cancer progression and metastasis	[341]
BLACAT1	EZH2/CDKN1C	Pancreatic cancer	BLACAT1 recruits EZH2 to provide trimethylation of CDKN1C promoter via H3K27 Enhancing proliferation and inducing glycolysis	[342]
HOTAIRM1	HOXA1/EZH2	Glioblastoma multiforme	Upregulation of HOXA1 by HOTAIRM1 Demethylation and sequestering EZH2 Increasing cancer proliferation and metastasis	[343]
LINC00115	TGF-β/LINC00115/EZH2	Glioma	MiRNA-200s down-regulation by TGF-β-mediated LINC00115 upregulation Inducing ZNF596 transcription Triggering EZH2/STAT3 axis for cancer progression	[344]
PVT1	EZH2/Hippo/Notch1	Non-small cell lung cancer	PVT1 stimulates Hippo/Notch1 axis via upregulating EZH2 Increasing cancer metastasis	[345]
FOXC2	EZH2/p15	Non-small cell lung cancer	Apoptosis inhibiting Preventing cell cycle arrest P53 down-regulation via activating EZH2 signaling	[346]
UCA1	EZH2/CDKN1A	Non-small cell lung cancer	Epigenetic silencing of CDKN1A via recruiting EZH2 Enhancing proliferation and inhibiting apoptosis	[347]
PVT1	MiRNA-526b/EZH2	Non-small cell lung cancer	Association with poor prognosis Down-regulation of miRNA-526b and subsequent induction of EZH2	[348]
MSTO2P	–	Lung cancer	Enhancing EZH2 expression and promoting proliferation and invasion of cancer cells	[228]
HOTAIR	–	Lung cancer	Silencing HOTAIR/EZH2 axis increases potential of atractylenolide 1 and erlotinib in lung cancer suppression	[349]
HOTAIR	–	Breast cancer	Apoptosis inhibition Inducing cell cycle progression Increasing cancer proliferation EZH2 recruitment Increasing DNA repair Inducing radio-resistance	[350]
TUG1	EZH2/miRNA-194-5p/CCND2	Bladder cancer	Recruiting EZH2 to down-regulate miRNA-194-5p Inhibiting CCND2 expression Silencing TUG1 increases cisplatin sensitivity of cancer cells	[351]
SPRY4-IT1	MiRNA-101-3p/EZH2	Bladder cancer	Increasing cancer proliferation and metastasis Reducing miRNA-101-3p expression via sponging Increasing EZH2 expression	[352]
AWPPH	EZH2/Smad4	Bladder cancer	Recruitment of EZH2 by AWPPH Subsequent upregulation of Smad4 and enhancing cancer proliferation and progression	[353]
CACS15	EZH2/APC	Ovarian cancer	Overexpression of CACS15 is associated with poor survival of cancer patients Increasing proliferation and metastasis Recruiting EZH2 to promoter of APC to inhibit it	[354]
SUMO1P3	EZH2/CPEB3	Colorectal cancer	Apoptosis inhibition and increasing proliferation upon SUMO1P3 overexpression Recruiting EZH2 to promoter of CREB3 Epigenetic repression of CREB3 by EZH2	[355]
DUXAP8	EZH2/LSD1	Colorectal cancer	DUXAP8 enhances EZH2 and LSD1 levels in providing cancer progression Association with tumor size and tumor grade	[356]
LL22NC03-N64E9.1	EZH2/KLF2	Colorectal cancer	Enhancing cancer proliferation and colony formation capacities Apoptosis inhibition Exerting carcinogenesis impact LncRNA binds to EZH2 to down-regulate KLF4 and provide its tumorigenesis impact	[357]
MALAT1	MiRNA-363-3p/EZH2	Colorectal cancer	Down-regulating miRNA-363-3p via sponging Inducing EZH2 signaling Enhancing cancer progression in vitro and in vivo	[358]
FAM83C	EZH2/SEMA3F	Colorectal cancer	Promoting malignant transformation of colorectal cancer Stabilizing EZH2 and increasing methylation of SEMA3F	[359]
SNHG6	MiRNA-26a/EZH2	Colorectal cancer	MiRNA-26a inhibition and subsequent upregulation of EZH2 Enhancing metastasis via EMT induction Increasing growth and survival of cancer cells	[341]
PRADX	EZH2/NF-κB	Colon adenocarcinoma	Recruiting EZH2 by PRADX in promoter of *UBXN1* gene NF-κB activation and increasing cancer progression	[360]
CASC11	STAT3/CASC11/EZH2/PTEN	Hepatocellular carcinoma	Overexpression of CASC11 by STAT3 Recruiting EZH2 and subsequent down-regulation of PTEN Enhancing cancer migration and invasion via EMT induction	[361]
HOXD-AS1	MiRNA-130a-3p/SOX4/EZH2	Liver cancer	Protecting SOX4 against degradation via miRNA-130a-3p down-regulation Enhancing EZH2 expression and paving the way for cancer progression	[362]
PVT1	EZH2/MYC	Liver cancer	Recruitment of EZH2 by PVT1 and subsequent induction of MYC expression Increasing cancer progression	[363]
SNHG8	EZH2/RECK	Cervical cancer	Apoptosis inhibition Facilitating proliferation Recruiting EZH2 for transcriptional repression of RECK	[364]
LINC01535	MiRNA-214/EZH2	Cervical cancer	Reverse relationship between LINC01535 and miRNA-214 Inducing EZH2 signaling Promoting growth in vitro and in vivo	[365]
PVT1	EZH2/miRNA-200b	Cervical cancer	Binding to EZH2 and recruiting it at promoter of miRNA-200b Enhancing proliferation and migration	[366]

### CircRNAs and EZH2 signaling

**Fig. 7:**
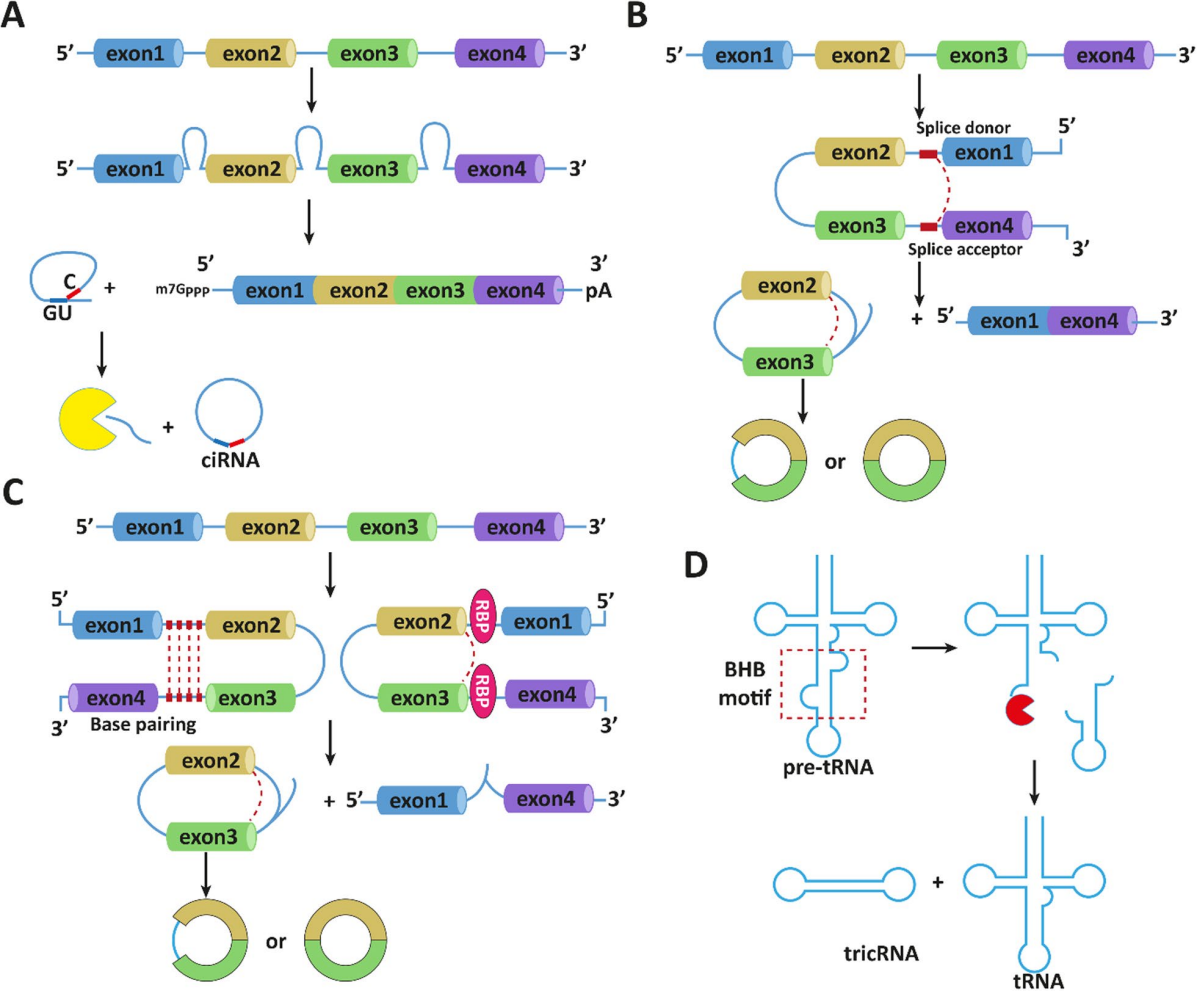
The circRNA biogenesis in cells [386]. **A** canonical splicing; **B** lariat-driven circularization; **C** intron-pairing-driven circularization; **D** bulge–helix–bulge (BHB) motif recognition

Circular RNAs (circRNAs) were first discovered in plant viroids and Sendai viruses in 1976 using electron microscopy [367]. Subsequently, attempts have been made to identify them in eukaryotic cells which occurred by 1979 [368–371]. However, it was believed that circRNAs result from splicing errors with low abundance [372]. Later, various kinds of circRNAs were discovered with progress in bioinformatics and sequencing technologies. CircRNAs have a loop structure attached through covalent bonds and lack 5′ cap and 3′ poly (A) tails (Fig. 7) [373]. The biogenesis of circRNAs starts with the precursor mRNA back-splicing of exons. In this way, a downstream 5/ splice site of an exon attaches to upstream 5/ splice site to form a loop structure [374–376]. Reverse complementary sequences and RNA binding proteins aid with exon skipping and circRNA formation [377–379]. It has been reported that expression of circRNAs is cell- and tissue-specific [380]. Recently, much attention has been directed towards the role of circRNAs in cancer as diagnostic, prognostic, and therapeutic tools [174, 381–384]. Furthermore, circRNAs can regulate miRNA expression via sponging like lncRNAs [385]. In this section, we provide a discussion of EZH2 regulation by circRNAs in cancer.

Most studies have focused on revealing the role of tumor-promoting circRNAs in cancer. Based on EZH2’s role in cancer progression, such circRNAs can enhance EZH2 expression. For instance, hsa-circ-0071589 undergoes upregulation in colorectal cancer cells and tissues. Mechanistically, this circRNA down-regulates miRNA-600 expression via sponging to enhance EZH2 expression [387]. Therefore, silencing such circRNAs may suppress tumorigenesis. For instance, silencing hsa-circ-0026123 is a promising strategy that enhances the expression level of miRNA-124-3p, leading to EZH2 down-regulation in ovarian cancer cells to inhibit their migration and growth [388]. In hepatocellular carcinoma, circ-LRIG3 enhances EZH2 expression and induces STAT3 methylation to promote cancer progression. Positive feedback loops between STAT3 and circ-LRIG3 occur through STAT3 binding to promoter of circ-LRIG3 and enhancing STAT3 expression, to subsequently lead to hepatocellular carcinoma progression [389]. These feedback loops are in favor of cancer malignancy and further complicates molecular pathways related to circRNA/EZH2 axis.

**Fig. 8:**
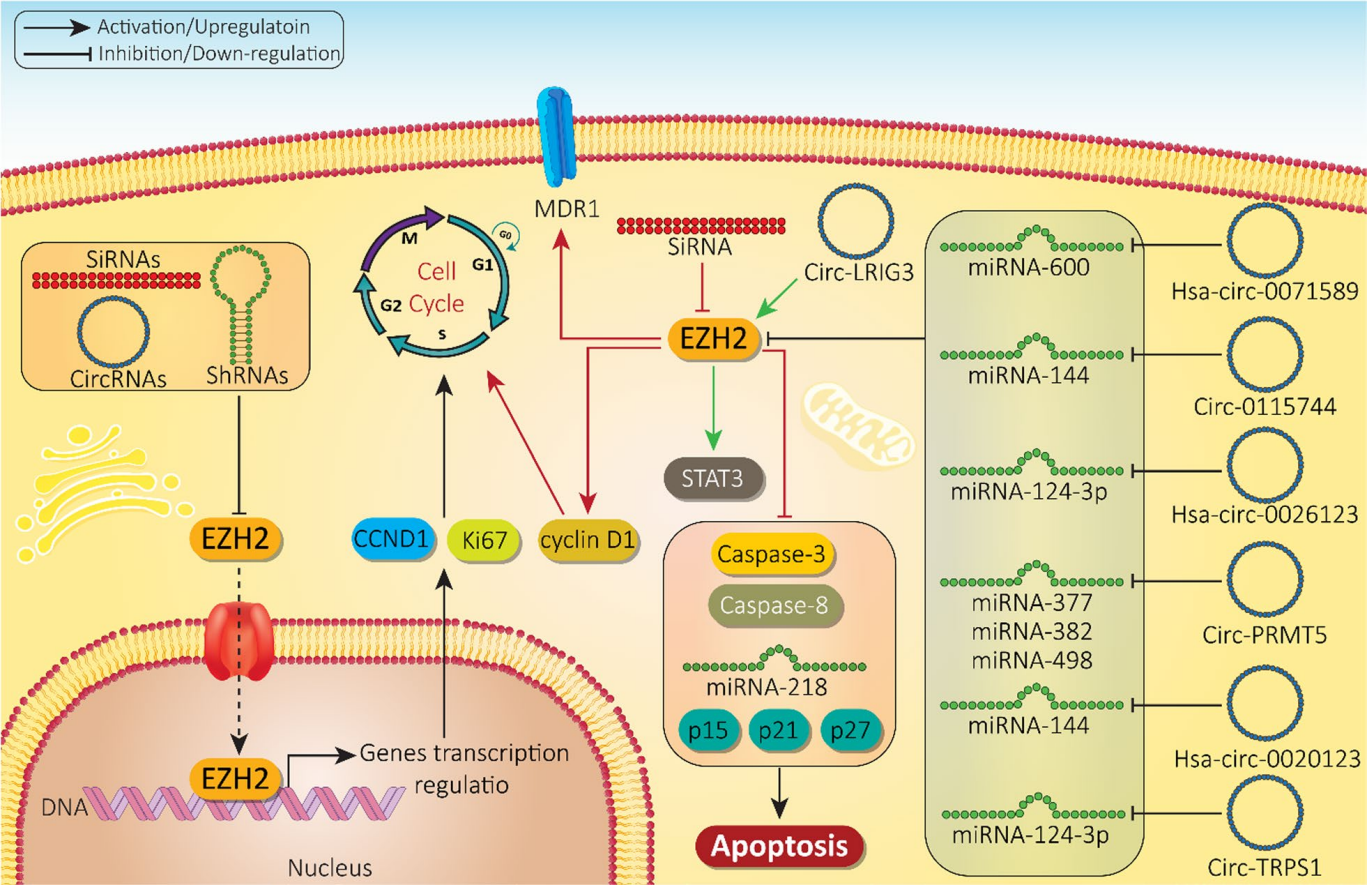
Regulation of EZH2 by siRNA, shRNA and circRNAs in cancer

New evidences demonstrate that circRNAs can target more than one miRNA when targeting EZH2. MiRNA-377, miRNA-382 and miRNA-498 are considered as tumor-suppressor factors in non-small cell lung cancer and their overexpression suppresses lung cancer growth via EZH2 down-regulation. On the other hand, circ-PRMT5 can down-regulate the expression levels of miRNA-377, -382 and -498 to enhance EZH2 expression, leading to an increase in proliferation of non-small cell lung cancer cells [390]. Therefore, competitive binding between circRNAs and miRNAs is of importance for regulating EZH2 expression and subsequent impact on proliferation and invasion of cancer cells [391]. One of the limitations of experiments related to circRNA/EZH2 axis is the lack of in vivo studies in many cancers. Most of studies have focused on in vitro models and we have still a long way before translation to clinic. It is noteworthy that the most the circRNAs investigated to date are tumor-promoting factors and their mechanism of action is suggested by sponging miRNAs to enhance EZH2 expression (Fig. 8 and Table 6) [392–394]. Further experiments need to focus on revealing the role of tumor-suppressor circRNAs in cancer.

**Table 6:** The role of CircRNAs in regulating EZH2 expression in different cancers

CircRNA	Signaling network	Cancer type	Signaling network	References
Hsa-circ-0071589	MiRNA-600/EZH2	Colorectal cancer	Reverse association between circRNA and miRNA-600 Reducing miRNA-600 expression via sponging Inducing EZH2 expression and promoting cancer progression	[387]
Circ-0115744	MiRNA-144/EZH2	Colorectal cancer	Reducing miRNA-144 expression and subsequent induction of EZH2 signaling Enhancing cancer metastasis	[392]
Hsa-circ-0026123	MiRNA-124-3p/EZH2	Ovarian cancer	Elevating cancer proliferation and migration Sponging miRNA-124-3p and increasing EZH2 expression	[206]
Circ-PRMT5	MiRNA-377/382/498/EZH2	Non-small cell lung cancer	Decreasing expression levels of miRNAs with tumor-suppressing role Paving the way for EZH2 upregulation Accelerating cancer progression	[390]
Hsa-circ-0020123	MiRNA-144/EZH2	Non-small cell lung cancer	Competitive binding with miRNA-144 and paving the way for EZH2 upregulation in elevating cancer progression	[391]
Circ-TRPS1	MiRNA-124-3p/EZH2	Prostatic cancer	Promoting stemness of cancer cells Reverse relationship between circRNA and miRNA Inducing EZH2 signaling	[393]
Circ-LRIG3	EZH2/STAT3	Hepatocellular carcinoma	Promoting expression level of STAT3 via activating EZH2 to ensure cancer survival	[389]
Hsa-circ-0000129	–	Breast cancer	Overexpression of EZH2 as a downstream target of circRNA Enhancing tumor progression Considering as a biomarker	[394]

## Non-coding RNA and EZH2 interaction in tumor microenvironment

The tumor microenvironment (TME) is a complex environment that not only comprises of cancer cells, abut also has immune cells, secreted proteins, adipocytes, fibroblasts and hematopoietic derived cells, among others. The interaction between cancer cells and TME affects and determines progression of tumor cells. The interactions occurring in TME are responsible for proliferation, invasion, angiogenesis and survival of cancer cells [395]. The EZH2 signaling affects TME to regulate cancer progression. For instance, EZH2 mutation affects infiltration of T cells in TME [396]. The EZH2 enhances viability and function of CD4 + and CD8 + T cells and can suppress differentiation of Th1 and Th2 cells [397]. The poor expression of EZH2 in CD8 + T cells leads to undesirable prognosis of cancers. Besides, EZH2 is responsible for preserving T-cell memory precursors in tumor suppression [398]. The T-regulatory cells, natural killer cells and dendritic cells are also affected by EZH2 signaling [399]. A recent experiment revealed that EZH2 upregulation by circPVT1 results in high infiltration of macrophages in TME and enhancing tumor progression [400]. Notably, immune checkpoint inhibitors are applied for improving cancer immunotherapy. However, resistance to immune checkpoint inhibitors has been observed in various tumors and EZH2 inhibition enhances anti-cancer immunity and prevents checkpoint inhibitor resistance via improving T regulatory cell trafficking and elevated antigen presentation [401].

The interaction between ncRNAs and EZH2 signaling in TME determines tumor progression. The down-regulation of miRNA-144/451a occurs in hepatocellular carcinoma and they are associated with anti-tumor immunity. This miRNA cluster negatively regulates EZH2 expression to elevate M1 polarization of macrophages and to enhance anti-tumor immunity. There is a negative feedback loop in which EZH2 can reduce miRNA expression in hepatocellular carcinoma to mediate immune evasion [402]. However, EZH2 functions in TME like a double-edged sword. The EZH2 signaling is vital for appropriate function of cytotoxic CD8 + T cells in TME and preventing tumor progression. The overexpression of miRNA-26a impairs function of T cells and promotes tumor progression, while miRNA-26a down-regulation is in favor of improving T cell function and inducing tumor growth suppression. The miRNA-26a has negative correlation with EZH2 in cytotoxic T cells and inhibits EZH2 signaling to disrupt T cell function and to enhance lung cancer progression [403]. An important feature of TME is hypoxia that can enhance both growth and metastasis of cancer cells [404, 405]. The expression level of lncRNA HITT undergoes down-regulation in hypoxic TME that promotes progression of cervical and colorectal tumor cells. The lncRNA HITT directs EZH2 to promoter of HIF-1α to form RNA–DNA triplex, leading to HIF-1α down-regulation and subsequent tumor suppression [406]. These studies highlight the fact that ncRNAs are capable of regulating EZH2 signaling in TME and affecting cancer progression.

## Biomarker application

As cancer claims the second leading cause of death after cardiovascular diseases, there have been efforts in identification of biomarkers for diagnosis, prognosis, and prediction. NcRNAs can be considered as reliable and non-invasive biomarkers for cancer diagnosis. It has been reported that upregulation of lncRNAs in lung cancer are associated transcript 1 (LUCAT1) which enhances cancer proliferation via EZH2 overexpression. Silencing LUCAT1 remarkably diminishes cancer progression and malignancy. With respect to its tumor-promoting role, LUCAT1 is considered as a prognostic factor as its upregulation provides undesirable prognosis for patients with thyroid cancer [407]. A similar phenomenon occurs in gastric cancer with lncRNA urothelial cancer-associated 1 (UCA1) which binds to EZH2 and induces the down-regulation of tumor-suppressor factors including p21 and Sprouty RTK signaling antagonist 1 (SPRY1). The lncRNA UCA1 is a prognostic factor in gastric cancer with its upregulation being correlated to unfavorable prognosis [408]. As another example, lncRNA SNHG1/EZH2 axis, a prognostic factor, contributes to rectal cancer metastasis and initiation [409]. These experiments highlight the fact that ncRNAs regulate EZH2 expression and can be considered as reliable and non-invasive biomarkers for cancer prognosis and diagnosis.

Pre-clinical studies are in agreement with the fact that EZH2 signaling is tightly regulated by ncRNAs. These findings are advantageous, when they are translated to clinic, paving the way for effective treatment of cancer patients. Throughout this review article, we report that lncRNAs, circRNAs and miRNAs can regulate EZH2 expression in cancer cells. It is noteworthy that most experiments have focused on revealing the role of tumor-promoting factors. It has been shown that siRNA and shRNA can be applied to down-regulate lncRNAs [410, 411]. Thus, next experiments can focus on the targeting of ncRNAs and translating these findings in clinical settings. Furthermore, as conditions in pre-clinical and clinical studies are different and elicit different responses in vivo and in vitro*,* strategies that increase the efficiency of therapeutics such as biocompatible, safe and well-tolerated nanocarriers for delivery of therapeutics in targeting ncRNA/EZH2 axis need be considered [412, 413]. Further experiments can help shed more light on the efficacy of nanocarriers at cellular and systemic levels.

## Conclusion and remarks

The present review provides a comprehensive discussion of EZH2 signaling role in cancer, and its regulation by upstream mediators, such as ncRNAs. The introduction section demonstrated the dual role of EZH2 in cancer. In most cases, EZH2 functions as a tumor-promoting factor, while owing to its methyltransferase activity, EZH2 may also function as tumor-suppressor. Tumor suppression occurs through recruitment of H3K27 to promoter of target genes and reducing their expression, paving the way for cancer elimination. Among ncRNAs, miRNAs and lncRNAs play a key regulatory impact on EZH2 signaling compared to other kinds of ncRNAs. MiRNAs can indirectly affect the expression of target gene by recruiting EZH2 to promoter. It is noteworthy that EZH2 can form a feedback and function as upstream mediator of miRNAs. In most cases, lncRNAs regulate the expression of EZH2 via sponging miRNAs. CircRNAs also regulate the expression of EZH2 signaling, but similar to lncRNAs, they regulate EZH2 expression mainly via targeting miRNAs. We also discuss clues for the development of siRNA and shRNA for targeting EZH2 and their clinical applications. However, efficacy of siRNA and shRNA is limited in vivo which will affect their potential in clinical course. Thus, we explored the use of nanocarriers for their targeted delivery in cancer patients which warrants additional research.

## Data Availability

Not applicable.

## Abbreviations

EZH2: Enhancer of zeste homolog 2
ncRNAs: Non-coding RNAs
PRCs: Polycomb repressive complexes
PRMT1: Protein arginine methyltransferase 1
EMT: Epithelial-to-mesenchymal transition
AOX1: Aldehyde oxidase 1
YAP: Yes-associated protein
CCL5: Chemokine ligand 5
miRNAs, microRNAs, 3′-UTR: 3′-Untranslated region
mRNA: Messenger RNA
RISC: RNA-induced silencing complex
LMO3: LIM-only protein 3
CSCs: Cancer stem cells
LC3: Light chain-3
STAT3: Signal transducer and activator of transcription 3
DOX: Doxorubicin
GKN1: Gastrokine 1
MMPs: Matrix metalloproteinases
IFN-γ: Interferon-γ
MALAT1: Metastasis-associated lung adenocarcinoma transcript 1
MVBs: Multivesicular bodies
PTEN: Phosphatase and tensin homolog
PTX: Paclitaxel
EN-2: Engrailed-2
GSK-3β: Glycogen synthase kinase-3beta
BDNF-AS: Brain-derived neurotrophic factor antisense
circRNAs: Circular RNAs
LUCAT1: Lung cancer associated transcript 1
CXCL12: Chemokine ligand 12
siRNA: Small interfering RNA
RNAi: RNA interference
MDR1: Multidrug resistance 1
shRNA: Short-hairpin RNA
lncRNAs: Long non-coding RNAs
SNHG1: Small nuclear RNA host gene 1
Akt: Protein kinase-B
PI3K: Phosphoinositide 3-kinase
PVT1: Plasmacytoma variant translocation I
TUG1: Taurine upregulated gene 1
JNK3: C-Jun N-terminal kinase 3
DZNeP: 3-Deacaneplanocin A
AR: Androgen receptor
ADT: Androgen deprivation therapy
HK2: Hexokinase-2
DNMT: DNA methyltransferases enzymes
MMP14: Matrix metalloproteinase 14
BRMS1: Breast cancer metastasis suppressor 1
CXCR4: CXC chemokine receptor 4
UCA1: Urothelial cancer-associated 1
SPRY1: Sprouty RTK signaling antagonist 1
