# Correction: The long and short non-coding RNAs modulating EZH2 signaling in cancer

**DOI:** 10.1186/s13045-022-01276-6

**Published:** 2022-05-06

**Authors:** Sepideh Mirzaei, Mohammad Hossein Gholami, Kiavash Hushmandi, Farid Hashemi, Amirhossein Zabolian, Israel Canadas, Ali Zarrabi, Noushin Nabavi, Amir Reza Aref, Francesco Crea, Yuzhuo Wang, Milad Ashrafizadeh, Alan Prem Kumar

**Affiliations:** 1 Department of Biology, Faculty of Science, Islamic Azad University, Science and Research Branch, Tehran, Iran; 2 Faculty of Veterinary Medicine, Kazerun Branch, Islamic Azad University, Kazerun, Iran; 3 Department of Food Hygiene and Quality Control, Division of Epidemiology and Zoonoses, Faculty of Veterinary Medicine, University of Tehran, Tehran, Iran; 4 Department of Comparative Biosciences, Faculty of Veterinary Medicine, University of Tehran, Tehran, 1417466191 Iran; 5 Department of Orthopedics, School of Medicine, 5Th Azar Hospital, Golestan University of Medical Sciences, Gorgan, Golestan Iran; 6 Blood Cell Development and Function Program, Fox Chase Cancer Center, Philadelphia, PA USA; 7 Department of Biomedical Engineering, Faculty of Engineering and Natural Sciences, Istinye University, Istanbul, 34396 Turkey; 8 Department of Urological Sciences and Vancouver Prostate Centre, University of British Columbia, Vancouver, BC V6H3Z6 Canada; 9 Belfer Center for Applied Cancer Science, Dana-Farber Cancer Institute, Harvard Medical School, Boston, MA USA; 10 Department of Translational Sciences, Xsphera Biosciences Inc., Boston, MA USA; 11 Cancer Research Group-School of Life Health and Chemical Sciences, The Open University, Walton Hall, Milton Keynes, MK7 6AA UK; 12 Faculty of Engineering and Natural Sciences, Sabanci University, Orta Mahalle, Üniversite Caddesi No. 27, Orhanlı, Tuzla, Istanbul 34956 Turkey; 13 Department of Pharmacology, Yong Loo Lin School of Medicine, National University of Singapore, Singapore, 117599 Singapore; 14 NUS Centre for Cancer Research (N2CR), Yong Loo Lin School of Medicine, National University of Singapore, Singapore, Singapore

## Correction: Journal of Hematology & Oncology (2022) 15:18 https://doi.org/10.1186/s13045-022-01235-1

The original article [1] contained an error in co-author, Farid Hashemi’s name which has since been corrected.
